# Control of fruit softening and Ascorbic acid accumulation by manipulation of *SlIMP3* in tomato

**DOI:** 10.1111/pbi.13804

**Published:** 2022-03-15

**Authors:** Xianzhe Zheng, Yujin Yuan, Baowen Huang, Xiaowei Hu, Yuwei Tang, Xin Xu, Mengbo Wu, Zehao Gong, Yingqing Luo, Min Gong, Xueli Gao, Guanle Wu, Qiongdan Zhang, Lu Zhang, Helen Chan, Benzhong Zhu, Zhengguo Li, Louise Ferguson, Wei Deng

**Affiliations:** ^1^ 47913 Key Laboratory of Plant Hormones and Development Regulation of Chongqing School of Life Sciences Chongqing University Chongqing China; ^2^ 7618 Department of Horticulture and Landscape Architecture Oklahoma State University Stillwater OK USA; ^3^ Department of Plant Sciences University of California Davis, One Shields Avenue Davis CA USA; ^4^ 34752 Laboratory of Fruit Biology College of Food Science & Nutritional Engineering China Agricultural University Beijing China

**Keywords:** fruit, softening, tomato, ascorbic acid, myoinositol

## Abstract

Postharvest deterioration is among the major challenges for the fruit industry. Regulation of the fruit softening rate is an effective strategy for extending shelf‐life and reducing the economic losses due postharvest deterioration. The tomato myoinositol monophosphatase 3 gene *SlIMP3*, which showed highest expression level in fruit, was expressed and purified. SlIMP3 demonstrated high affinity with the L‐Gal 1‐P and D‐Ins 3‐P, and acted as a bifunctional enzyme in the biosynthesis of AsA and myoinositol. Overexpression of *SlIMP3* not only improved AsA and myoinositol content, but also increased cell wall thickness, improved fruit firmness, delayed fruit softening, decreased water loss, and extended shelf‐life. Overexpression of *SlIMP3* also increased uronic acid, rhamnose, xylose, mannose, and galactose content in cell wall of fruit. Treating fruit with myoinositol obtained similar fruit phenotypes of *SlIMP3‐*overexpressed fruit, with increased cell wall thickness and delayed fruit softening. Meanwhile, overexpression of *SlIMP3* conferred tomato fruit tolerance to *Botrytis cinerea*. The function of *SlIMP3* in cell wall biogenesis and fruit softening were also verified using another tomato species, Ailsa Craig (AC). Overexpression of *SlDHAR* in fruit increased AsA content, but did not affect the cell wall thickness or fruit firmness and softening. The results support a critical role for *SlIMP3* in AsA biosynthesis and cell wall biogenesis, and provide a new method of delaying tomato fruit softening, and insight into the link between AsA and cell wall metabolism.

## Introduction

By value, tomato is the fourth most important commercial crop globally (Vincent *et al*., 2013). Tomato is a rich source of minerals, vitamins, and phytochemicals. Postharvest deterioration is among the major challenges for fruit industry, accounting for up to 50% of harvested losses (Thole *et al*., 2020). The primary cause of postharvest deterioration is fruit softening, which decreases fruit shelf‐life and increases susceptibility to pathogens (Brummell and Harpster, 2001). Theoretically, regulating the rate of softening would extend shelf‐life and increase pathogen resistance and be an effective strategy to reduce postharvest losses (Uluisik *et al*., 2016).

Fruit softening is result of destruction of the fruits wall’s structural polysaccharides and reduction in intercellular cell wall adhesion (Martin *et al*., 2014; Seymour *et al*., 2013). The main components of the cell wall include cellulose, hemicellulose, pectin, and a small amount of protein (Bashline *et al*., 2014). Due to the complex composition and structure, many enzymes have been reported to catalyse the fruit softening. The role of polygalacturonase (PG), pectin methyl esterase (PME), β‐galactanase, expansin, and pectate lyases (PLs) regulating fruit texture has been well investigated. Downregulation of the *PG* and *PME* genes does not affect tomato fruit softening (Hall *et al*., 2010; Sheehy *et al*., 1988; Smith *et al*., 1988, 1990; Tieman and Handa, 1994; Tieman *et al*., 1992). Silencing of the β‐galactanase and expansin genes has a moderate effect on fruit softening (Brummell *et al*., 1999; David *et al*., 2002). Silencing the *PL* gene in tomato delayed fruits softening and reduced susceptibility to grey mould, implying prolonging fruit shelf‐life by genetic modification of cell wall‐modifying enzymes is a potential approach (Uluisik *et al*., 2016; Yang *et al*., 2017).

Ascorbic acid (AsA), vitamin C, a crucial compound is present in most living organisms (Laing *et al*., 2015). In higher plants, AsA functions as an antioxidant and enzymatic cofactor, playing a crucial role in multiple physiological processes including photoprotection, cell expansion and division, ethylene biosynthesis and abiotic stress responses (Alhagdow *et al*., 2007; Hu *et al*., 2016). As a result of these critical functions in plants and its benefits to human health, AsA biosynthesis, recycling, and accumulation in plants have been extensively investigated. The current consensus is that the L‐galactose pathway is the primary pathway for AsA accumulation in higher plants. The structural genes have been identified. L‐galactose 1‐phosphate phosphatase (GPP) catalyses the conversion of L‐galactose 1‐phosphate (L‐Gal 1‐P) to L‐galactose in AsA synthesis (Torabinejad *et al*., 2009). It has been reported that the expression patterns of *GPP* are associated with AsA content in apple and tomato plants under abiotic stress (Ioannidi *et al*., 2009; Li *et al*., 2010). In Arabidopsis, the *VTC4* gene (At3g02870) encodes an enzyme catalysing the similar reaction with the GPP enzyme in AsA biosynthesis (Valpuesta and Botella, 2005). The VTC4 gene has been reported to be a bifunctional enzyme, also catalysing conversion of D‐myoinositol 3‐phosphate (D‐Ins 3‐P) to myoinositol in myoinositol biosynthesis (Torabinejad *et al*., 2009). The myoinositol can be converted to UDP‐glucuronic acid (UDP‐GlcA), which is a common but cell wall‐specific biochemical precursor for cell wall biogenesis (Loewus, 2006; Loewus and Murthy, 2000; Reiter, 2003). The bifunctional VTC4 enzyme facilitates formation of AsA and cell walls (Figure S1). Therefore, the *VTC*4 may be a candidate gene for enhancing nutrition and delaying softening via influencing of the AsA production and cell wall formation in tomato fruit.

Tomato has three *VTC4* homologue genes *SlIMP1*, *SlIMP2,* and *SlIMP3* (Gillaspy *et al*., 1995). The *SlIMP3*, which has the highest expression level in tomato fruit, was selected for this investigation as it also has bifunctional enzyme activity, similar to that of the VTC4 in Arabidopsis. Overexpression of *SlIMP3* in tomato increases the AsA content in multiple tissues. Overexpression of *SlIMP3* increased the myoinositol accumulation, cell wall thickness, and altered cell‐wall composition. Overexpression of *SlIMP3* markedly delayed fruit softening and enhanced fruit resistance to *Botrytis cinerea*. The results demonstrate a critical role for *SlIMP3* in AsA biosynthesis and cell wall biogenesis and provide new method of delaying fruit softening and extending shelf‐life of tomato.

## Results

### Sequence analysis and expression profiles of SlIMP3 in tomato

**Figure 1:**
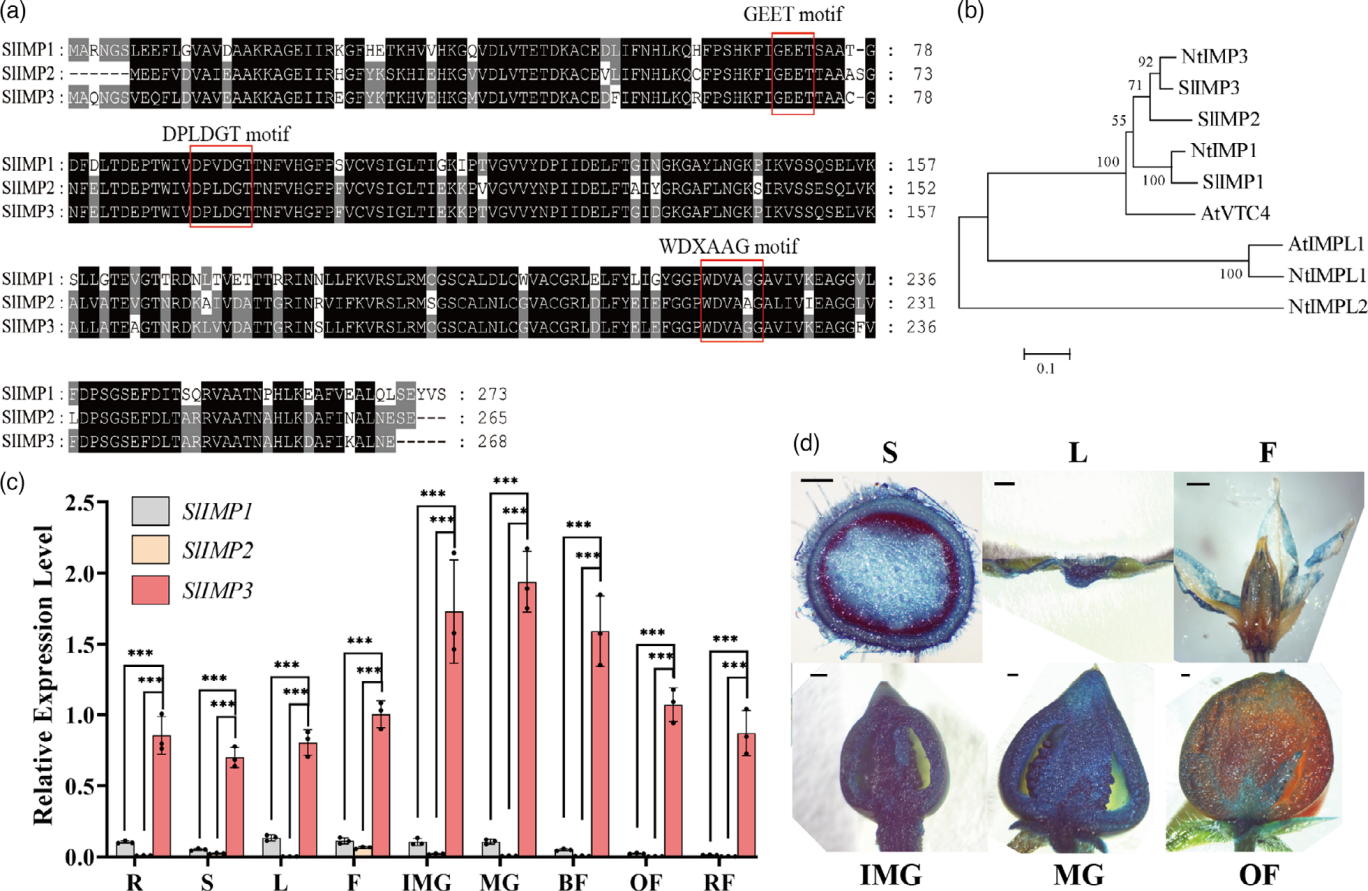
Protein structure and expression analysis of SlIMP3. (a) Protein sequences alignment and conserved domain analysis of SlIMP1, SlIMP2, and SlIMP3. (b) Phylogenetic tree of IMPs proteins in Arabidopsis thaliana (At), Nicotiana tabacum (Nt), and Solanum lycopersicum (Sl) constructed by the neighbour‐joining method. (c) Differences in relative expression level of *SlIMPs* in various tomato tissues: R root, S shoot, L leaf, F flower, IMG immature green fruit, MG mature green fruit, BF break fruit, OF orange fruit, RF red fruit. All data contain three replicates, and the standard deviation (SD) is given by error bar. Statistical significance between *SlIMP3* and *SlIMP1/SlIMP2* was indicated by asterisk (***P < 0.001). (d) GUS staining of ProSlIMP3::GUS transgenic plants in different tissues: S shoot, L leaf, F flower, IMG immature green fruit, MG mature green fruit, OF orange fruit. Scale bars, 1 mm.

Three *IMP* genes have been identified in tomato (Gillaspy *et al*., 1995). Alignment and sequence analysis revealed that the IMP amino acid sequences contained signature motifs (DPLDGT, WDXAAG, and GEET) (Figure 1a). A phylogenetic analysis of tomato IMP sequences, together with Arabidopsis and tobacco IMP‐related genes, was carried out using the neighbour‐joining method on mega6. The results indicated that *SlIMP3* was most closely related to *NtIMP3* and clustered with *SlIMP1*, *NtIMP1,* and *AtVTC4* into one subfamily (Figure 1b). The expression pattern of three *SlIMPs* in vegetative and reproductive tissues was carried out using the online TomExpression platform. The *SlIMPs* had ubiquitous expression in all tested tissues, including roots, shoots, leaves, flowers, and fruits. It was interesting that *SlIMP3* gene had highest expression levels during fruit development and ripening (Figure S2). A qRT‐PCR test was performed to confirm the expression patterns of *SlIMPs* in tomato plants. The results were consistent with the TomExpression data, with the highest expression levels of *SlIMP3* in fruits (Figure 1c). The expression pattern of *SlIMP3* was also explored through a transgenic tomato plant in which GUS reporter gene was driven by the *SlIMP3* promoter. Consistent with the qRT‐PCR results, the GUS staining revealed the ubiquitous expression pattern of *SlIMP3* in leaves, stems, buds, flowers, and fruits at different developmental stages, with strong expression in immature green fruit. The expression of *SlIMP3* was also decreased in the ripening stages, though weakly expression in orange fruits (Figure 1d).

### The catalytic properties of SlIMP3 enzyme are similar with Arabidopsis VTC4 and *SlIMP3* gene regulates AsA biosynthesis in Micro‐tom tomato

**Figure 2:**
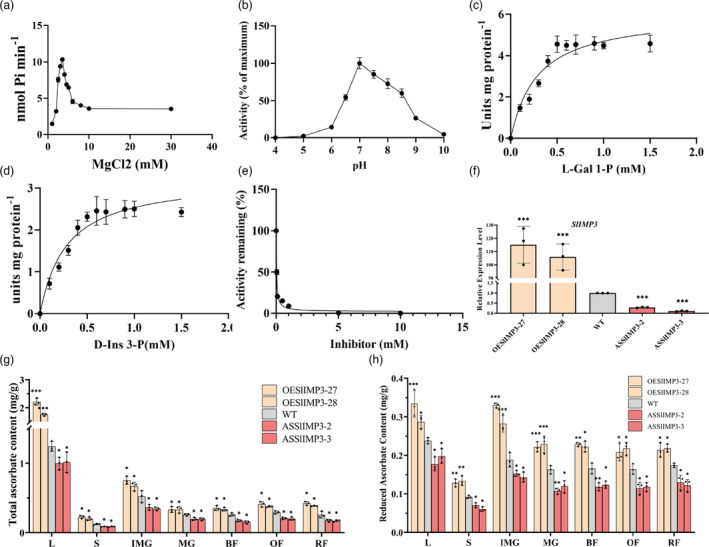
SlIMP3 activity analysis and positive regulation of AsA biosynthesis. (a) Enzyme activity at different magnesium concentrations. A 50 μL of mixture with 50 mm of Tris‐Cl, pH 7.0, 0.5 mm of D‐Ins 3‐P, 2 mg of purified SlIMP3 proteins, and varying concentrations of MgCl_2_ was used as standard conditions. (b) Enzyme activity at different pH values. Except for the different pH value and the determined 3.5 mm of MgCl_2_, the other conditions are the same as (a). (c, d) Substrate saturation curve of SlIMP3 at different L‐Gal 1‐P (a) and D‐Ins 3‐P (b) concentrations. K_m_ and V_max_ were calculated according to Michaelis–Menten equation by GraphPad Prism 8 software. (e) Inhibition of SlIMP3 activity by LiCl. IC50 were calculated according to substrate inhibition equation by GraphPad Prism 8 software. (f) Relative expression level of *SlIMP3* in overexpression (OESlIMP3‐27, OESlIMP3‐28) and antisense (ASSlIMP3‐2, ASSlIMP3‐3) lines. (g, h) Total ascorbate and reduced ascorbate contents in different tomato tissues (L leaf, S stem, IMG immature green fruit, MG mature green fruit, BF break fruit, OF orange fruit, RF red fruit) of WT, OESlIMP3, and ASSlIMP3 lines. All data contain three replicates and SD was given by error bar. Statistical significance between WT and OESlIMP3/ASSlIMP3 lines was indicated by asterisk (*P < 0.05, **P < 0.01, ***P < 0.001).

It is established that the *VTC4* gene encodes a bifunctional enzyme that influences myoinositol and ascorbate biosynthesis (Torabinejad *et al*., 2009). We expressed and purified recombinant SlIMP3 to analyse the catalytic character. The open reading frame of the *SlIMP3* gene was cloned into a pGEX‐4T‐1 vector to generate translational fusion with glutathione‐S‐transferases (GSTs). The recombinant protein was expressed in *E. coli* and purified with affinity chromatography. The molecular mass of the GST‐SlIMP3 fusion protein was estimated to be 55 kD (Figure S3), similar to the predicted molecular mass. Mg^2+^ is necessary for myoinositol monophosphatase activity. Optimal SlIMP3 activity was obtained by examining the optimum MgCl_2_ concentration for enzyme activity from 1 to 30 mm of MgCl_2_. The 3.5 mm of MgCl_2_ concentration was the most effective (Figure 2a) at a pH of 7.0 (Figure 2b).

The Arabidopsis VTC4 enzyme has been reported to use L‐Gal 1‐P and D‐Ins 3‐P as substrates (Torabinejad *et al*., 2009). Therefore, the catalytic properties of SlIMP3 enzyme were analysed using L‐Gal 1‐P and D‐Ins 3‐P as substrates. In the reaction mixtures of 3.5 mm of MgCl_2_, pH 7.0, and 2 mg of enzyme, the apparent K_m_ for L‐Gal 1‐P was 0.29 mm and that for D‐Ins 3‐P was 0.28 mm (Figure 2c,d). The apparent Vmax values of SlIMP3 for L‐Gal 1‐P and D‐Ins 3‐P calculated were 6.0 and 8.0 units, respectively.

LiCl was an important inhibitor, which inhibited the catalytic effect of IMPs on the substrate. In previous studies, LiCl inhibited the catalytic activity of VTC4 for D‐Ins 3‐P, and the half‐maximal inhibitory concentration (IC50) was 0.08 to 0.1 mm (Torabinejad *et al*., 2009). The inhibition of SlIMP3 for D‐Ins 3‐P by LiCl was also tested (Figure 2e). The IC50 of SlIMP3 with LiCl was 0.03 to 0.05 mm when the reaction contained 0.5 mm of substrate.

The relationship between AsA biosynthesis and *SlIMP3* gene function, transgenic lines expressing either sense or antisense *SlIMP3* constructs under the control of the cauliflower mosaic virus 35S promote were further characterized. A qRT‐PCR was conducted to analyse the expression levels. The independent overexpressed or antisense lines, which displayed substantial altered gene expression by comparison with the wild type (WT) plants, were used for further analysis (Figure 2f).

Total AsA and reduced AsA content quantification were conducted using these transgenic plants. Interestingly that altered *SlIMP3* expression led to dramatic AsA content change in different tissues of the transgenic lines (Figure 2g,h). The AsA content quantification in leaf, stem, and different fruit developmental stages indicated that *SlIMP3*‐overexpressed fruits accumulated higher amounts of AsA than the WT plants, whereas the downregulated fruits had lower AsA content than the WT plants. In addition, qRT‐PCR results indicated that *SlPGI*, *SlGMP1*, *SlGMP3*, *SlGGP1*, *SlGGP2,* and *SlGalLDH* were significantly up‐regulated in *SlIMP3*‐overexpressed fruits (Figure S4). We also found that *SlGalLDH* was significantly down‐regulated in downregulated fruits (Figure S4). These results indicated that the *SlIMP3* gene regulated AsA biosynthesis in tomato plants.

### Overexpression of *SlIMP3* delays fruit softening and increases cell wall thickness in Micro‐tom tomato

**Figure 3:**
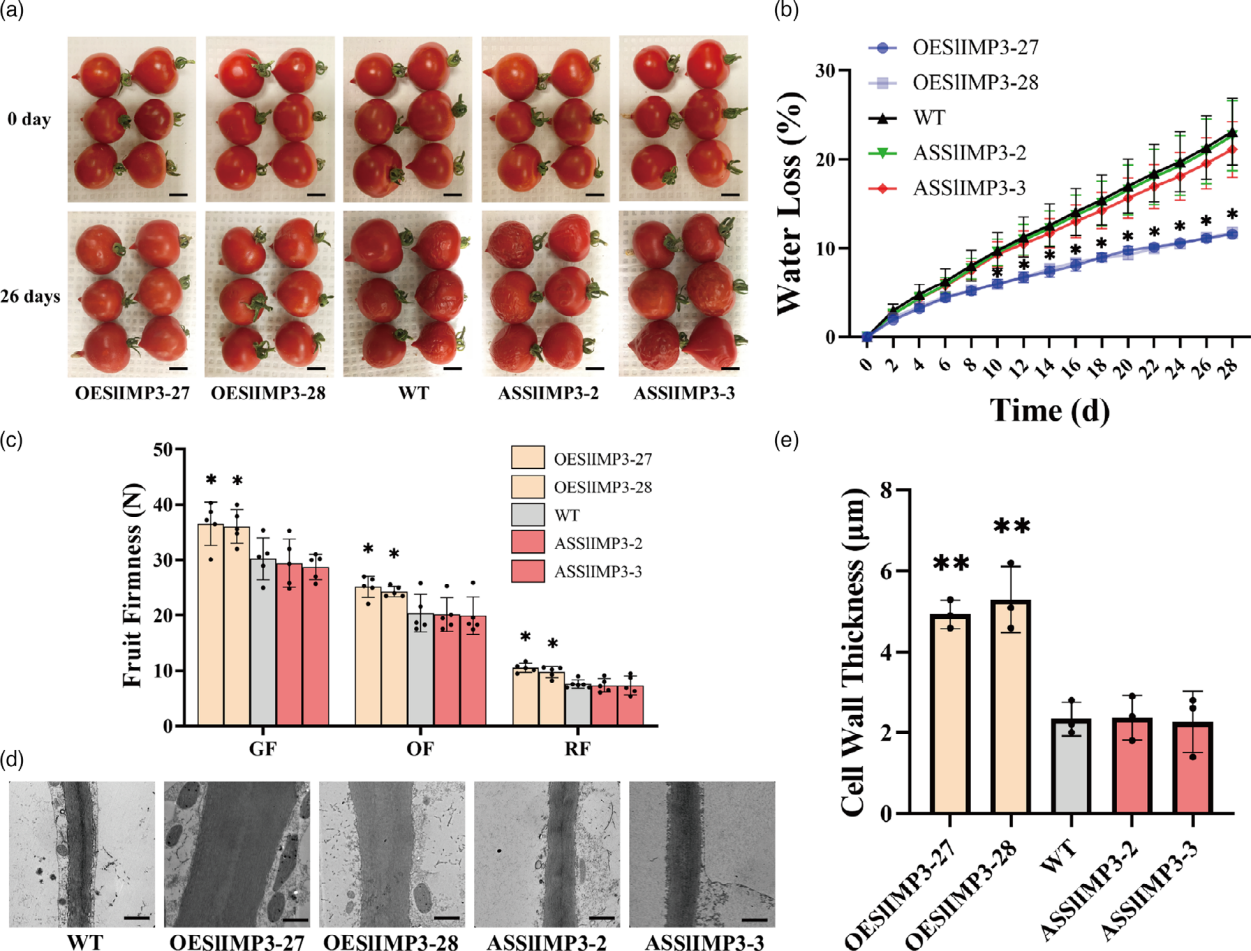
Overexpression of SlIMP3 delays fruit softening and increased cell wall thickness in ‘Micro‐tom’ tomato. (a) Red fruits of WT, OESlIMP3, and ASSlIMP3 lines were stored at 25°C for 0 and 26 days. Scale bars, 5 mm. (b) Water loss rate of fruits in WT, OESlIMP3, and ASSlIMP3 lines. Statistics of water loss rate for every two days. The data contain four replicates: the error bar represents the SD. (c) Firmness of fruits at different developmental stages: GF green fruit, OF orange fruit, RF red fruit. (d, e) The difference in cell wall thickness between WT and OESlIMP3/ASSlIMP3 lines. The cell wall of fruit pericarp cells was observed with transmission electron microscope. The cell wall thickness was measured with ImageJ software. The data contain three replicates and SD was given by error bar. Scale bars, 1 μm. Statistical significance between WT and OESlIMP3/ASSlIMP3 lines was indicated by an asterisk (*P < 0.05, **P < 0.01).

Postharvest fruit storage was conducted to test the influence of *SlIMP3* overexpression on fruit softening. The WT and SlIMP3‐downregulated fruits were shrivelled after 26 d of storage versus the well maintained pericarp quality of the SlIMP3‐overexpressed fruits (Figure 3a). The fruit water loss of the SlIMP3‐overexpressed fruits was less than that of WT and SlIMP3‐downregulated fruits (Figure 3b). The *SlIMP3‐*overexpressed fruits also were firmer throughout fruit development versus the downregulated fruits and WT fruits, which were again similar (Figure 3c). Ethylene production in *SlIMP3*‐overexpression and *SlIMP3*‐downregulated fruits did not change significantly compared with the WT fruits (Figure S5). Overexpression of *SlIMP3* had no effect on fruit yield and weight (Figure S6). In addition, both the up‐regulation and down‐regulation of *SlIMP3* did not affect the fruit development and maturation (Table S1).

Transmission electron microscopy (TEM) was used to examine the cell wall thickness to determine the effects of altered *SlIMP3* expression on cell walls. The *SlIMP3*‐overexpression fruits had thicker cell walls than those of WT fruit but not of those of *SlIMP3*‐downregulation fruits (Figure 3d,e). At the same time, we measured the expressions of cell wall metabolism‐related genes as previously described (Zhang *et al*., 2018). qRT‐PCR results indicated that *SlEXP1*, *SlPG2*, *SlPL*, *SlTBG4*, *SlXYL1,* and *SlXTH5* were significantly down‐regulated in *SlIMP3*‐overexpressed fruits (Figure S7). Moreover, like fruits, the cell wall in *SlIMP3*‐overexpression leaves and stems were also increased (Figure S9). Collectively, these results suggested overexpression of *SlIMP3* increased the cell wall thickness, delayed the fruit softening, and enhanced tomato shelf‐life.

### Overexpression of *SlIMP3* and myoinositol treatment increase cell wall biogenesis in fruit of ‘Micro‐Tom’ tomato

**Figure 4:**
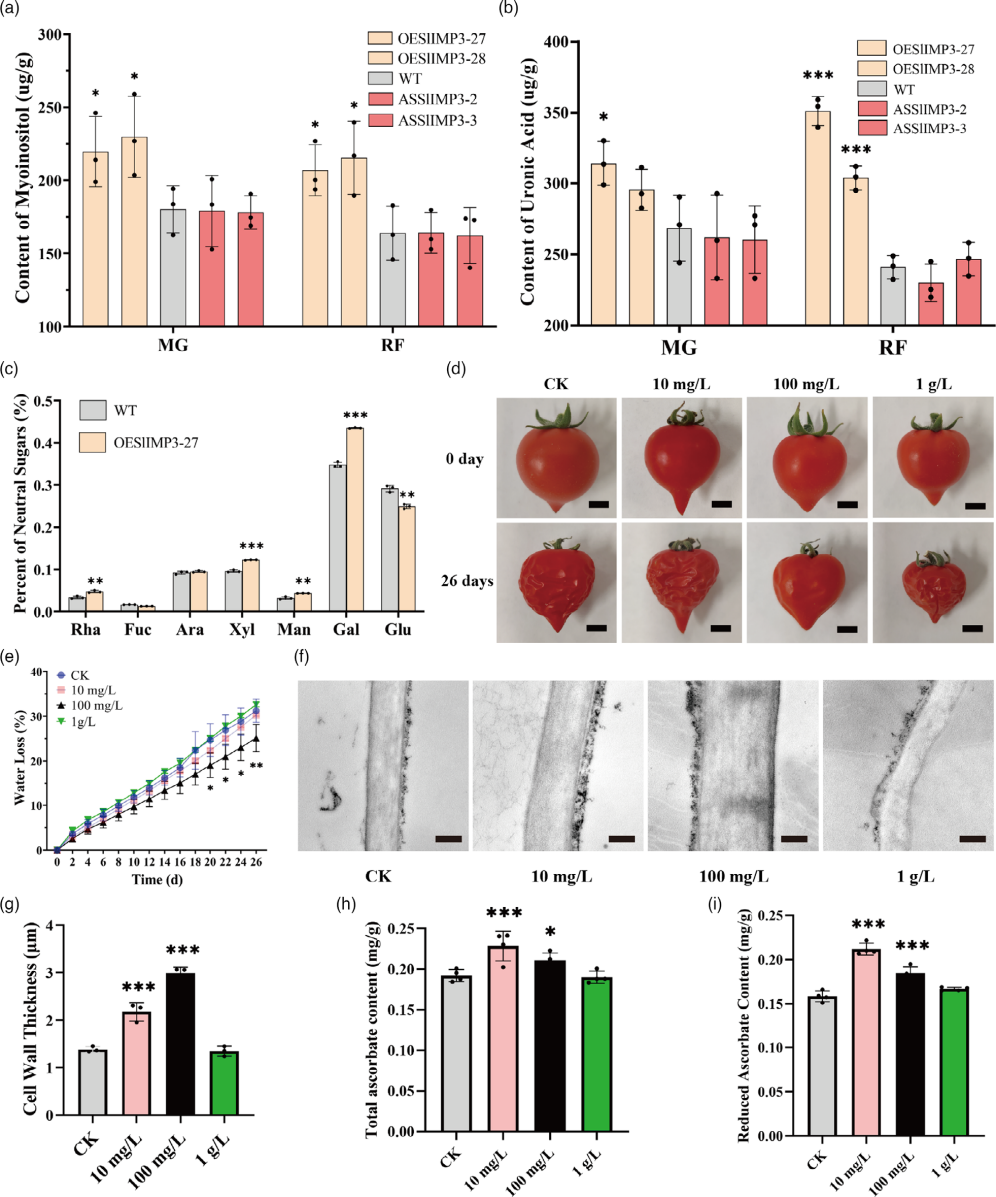
Cell wall composition analysis and effects of exogenous myoinositol treatment on fruit softening. (a, b) Content of myoinositol (a) and uronic acid (b) of fruits in WT, OESlIMP3, and ASSlIMP3 lines at different developmental stages: MG mature green fruit, RF red fruit. (c) Percent of neutral sugars (Rha rhamnose, Fuc fucose, Ara arabinose, Xyl xylose, Man mannose, Gal galactose, and Glu glucose) of WT, OESlIMP3, and ASSlIMP3 lines fruits at red fruit stage. (d) Red fruits of CK (control check) and myoinositol treatment lines were stored at 25°C for 0 day and 26 days. Scale bars, 5 mm. Statistics of water loss rate every two days. All data contain three replicate and SD was given by error bar. Statistical significance between WT and OESlIMP3/ASSlIMP3 lines was indicated by an asterisk (*P < 0.05, **P < 0.01, ***P < 0.001). (e) Water loss rate of CK and myoinositol treatment lines. (f, g) Cell wall thickness in CK and myoinositol treatment lines. Scale bars, 1 μm. The cell wall of fruit pericarp cells was observed by transmission electron microscope, and the thickness of the cell wall was measured with ImageJ software. The data contain three replicates; the error bars represent the SD. Scale bars, 1 μm. (h, i) Total ascorbate and reduced ascorbate contents in CK and myoinositol treatment lines. All data contain three replicates and SD was given by error bar. Statistical significance between CK and myoinositol treatment lines was indicated by an asterisk (*P < 0.05, **P < 0.01, ***P < 0.001).

To gain more insight into the mechanism by which cell wall thickness was impacted in *SlIMP3*‐overexpressed plants, biochemicals related to cell wall biogenesis was analysed. Measurement demonstrated overexpression of the *SlIMP3* gene increased myoinositol in mature green and red fruits versus no effect of the downregulation of *SlIMP3* gene (Figure 4a). Uronic acid content was measured in the cell wall of mature green and red fruits by high‐performance liquid chromatography (HPLC). The uronic acid content increased markedly in *SlIMP3*‐overexpressed red fruits but was unchanged in *SlIMP3*‐downregulated fruits and WT fruits (Figure 4b). Various neutral sugars, rhamnose, fucose, arabinose, xylose, mannose, galactose, and glucose were also measured. The rhamnose, xylose, mannose, and galactose content of *SlIMP3‐*overexpressed red fruits were significantly higher than that of WT (Figure 4c). Collectively, these results suggested that overexpression of *SlIMP3* improved cell wall biogenesis in tomato fruit.

Because myoinositol is related to cell wall biogenesis, the WT tomato plants were treated with 10 mg/L, 100 mg/L, and 1 g/L of myoinositol to examine the role myoinositol in cell wall biogenesis and fruit softening. When treated with 10 mg/L of myoinositol, the fruit cell wall thickness increased slightly, but the fruit softening and water loss were unchanged versus the control. When treated with 100 mg/L, myoinositol fruit softening was delayed, cell wall thickness increased, and water loss decreased significantly (Figure 4d–g). However, after treatment with 1 g/L of myoinositol, fruit cell wall thickness, storage life, and water loss were unaffected (Figure 4d–g). The 10 mg/L and 100 mg/L of myoinositol treatments sharply increased fruit AsA concentration, but 1 g/L of myoinositol did not modulate fruit AsA production (Figure 4h, i). These experimental results demonstrated exogenous myoinositol application 100 mg/L enhanced the cell wall biogenesis and delayed softening in tomato fruits.

### Overexpression of *SlIMP3* in Micro‐tom tomato increases the tolerance of fruit to *Botrytis cinerea*

**Figure 5:**
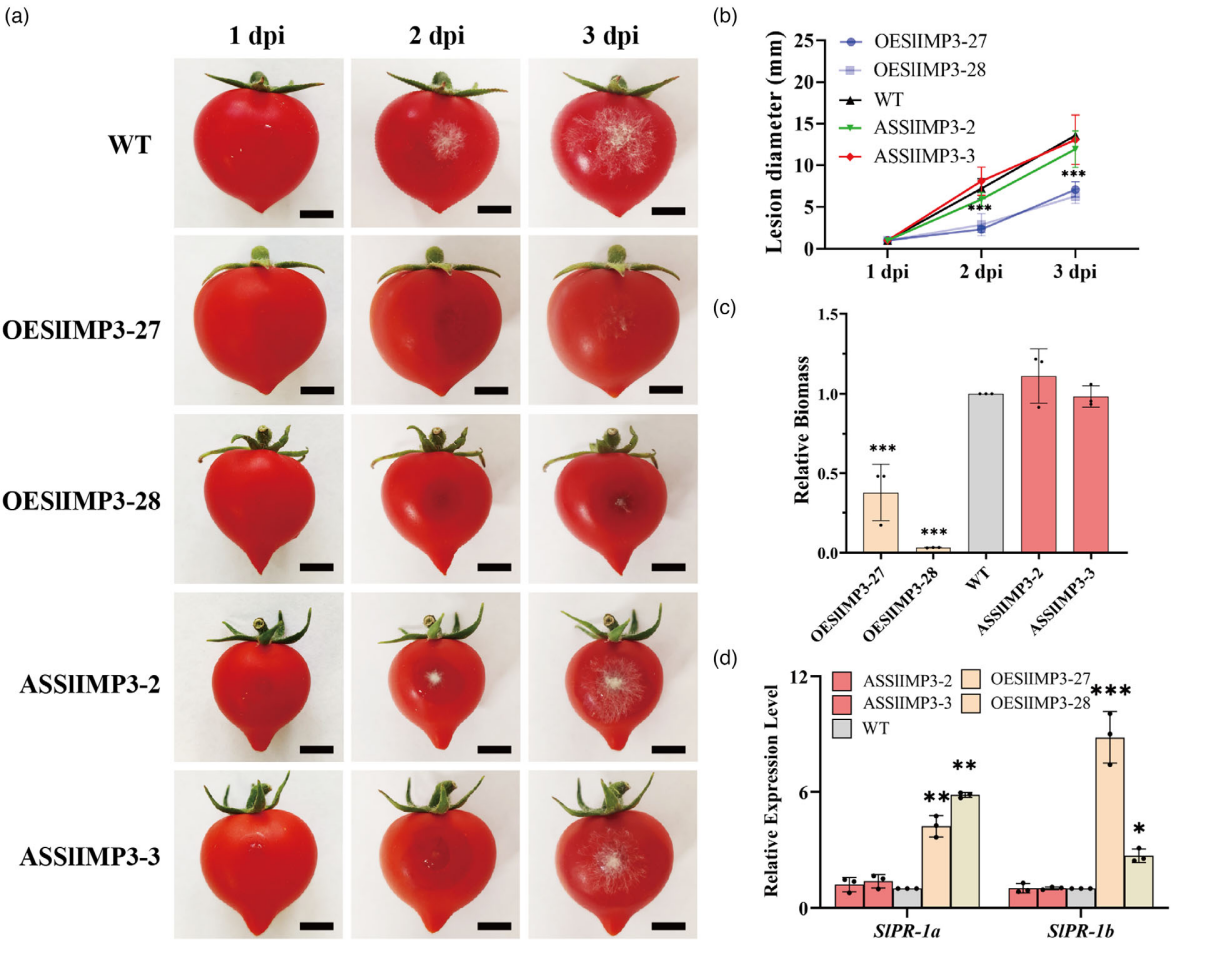
Overexpression of SlIMP3 increased the tolerance of fruit to *Botrytis cinerea*. (a) Symptoms of wounded WT and OESlIMP3/ASSlIMP3 lines fruits after been infested by *Botrytis cinerea* at 1 dpi, 2 dpi, and 3 dpi. (b) Lesion diameter was measured by ImageJ software at 1 dpi, 2 dpi, and 3 dpi. (c) The biomass of *Botrytis cinerea* in WT and OESlIMP3/ASSlIMP3 lines fruits was determined by qRT‐PCR. (d) Relative expression level of pathogen‐related genes (*SlPR‐1a* and *SlPR‐1b*) in WT and OESlIMP3/ASSlIMP3 lines fruits was determined by qRT‐PCR. All data contain three replicates and SD was given by error bar. Statistical significance between WT and OESlIMP3/ASSlIMP3 lines was indicated by an asterisk (*P < 0.05, **P < 0.01, ***P < 0.001).

*Botrytis cinerea* (*B. cinerea*) is a major pathogen causing tomato losses during postharvest storage (Xiong *et al*., 2019; Yang *et al*., 2017). A *B. cinerea* spore suspension surface applied to injured tomato transgenic tomato fruits produced significantly smaller lesion diameters in *SlIMP3*‐overexpressed fruits versus those in WT and *SlIMP3*‐downregulated fruits, which were approximately equal (Figure 5a,b). Similarly, the biomass of *B. cinerea* detected with qRT‐PCR was significantly lower in the *SlIMP3*‐overexpressed fruits versus the WT and downregulated fruit, which were again approximately equal (Figure 5c). Moreover, the expression levels of pathogen‐related genes (*SlPR‐1a* and *SlPR‐1b*) were significantly up‐regulated in *SlIMP3*‐overexpressed fruits but were unchanged in *SlIMP3*‐downregulated fruits and WT fruits (Figure 5d). This demonstrated overexpression *SlIMP3* in tomato significantly improved the tolerance to *B. cinerea*.

### Overexpression of *SlIMP3* gene increases cell wall thickness and delays fruit softening in ‘Ailsa Craig’ tomato

**Figure 6:**
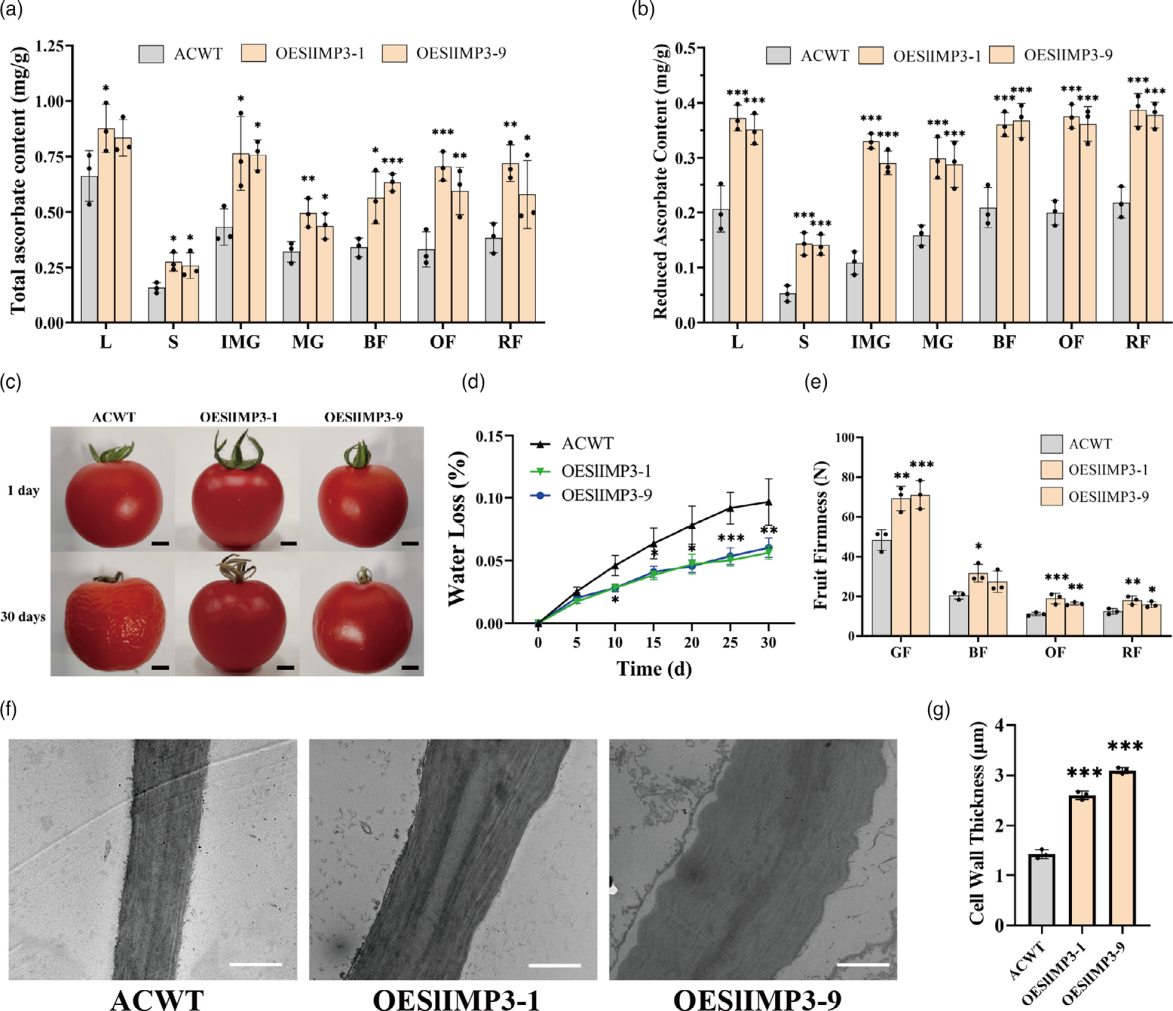
Overexpression of SlIMP3 gene increased cell wall thickness and delayed fruit softening in Ailsa Craig tomato. (a, b) Total ascorbate and reduced ascorbate contents in different tomato tissues (L leaf, S stem, IMG immature green fruit, MG mature green fruit, BF break fruit, OF orange fruit, RF red fruit) of ACWT (Ailsa Craig wild type) and overexpression (OESlIMP3‐1, OESlIMP3‐9) lines. (c) Red fruits of ACWT and OESlIMP3‐1 lines were stored at 25°C for 0, 8, 15 and 30 days. Scale bars, 5 mm. (d) Water loss rate of ACWT and OESlIMP3‐1 lines. Statistics of water loss rate every five days. (e) Firmness of fruits at different developmental stages: GF green fruit, BF break fruit, OF orange fruit, RF red fruit. (f, g) The difference in cell wall thickness between ACWT and OESlIMP3 lines. The cell wall of fruit pericarp cells was observed by transmission electron microscope, and the cell wall thickness was measured with ImageJ software. Scale bars, 1 μm. All data contain three replicates and SD was given by error bar. Statistical significance between ACWT and OESlIMP3 lines was indicated by an asterisk (*P < 0.05, **P < 0.01, ***P < 0.001).

The function of *SlIMP3* in cell wall formation and fruit softening was examined in the tomato cultivar ‘Ailsa Craig’ (AC). The same *SlIMP3*‐overexpressed construct was transformed into ‘AC’ tomato via *Agrobacterium tumefaciens‐*mediated transformation. The qRT‐PCR results demonstrated the *SlIMP3* expression level was significantly increased in the overexpression lines compared with the WT plants (Figure S10). The total and reduced AsA contents in leaf, stem, and in the different developmental fruit stages of the *SlIMP3*‐overexpressed plants were higher than those of the WT plants (Figure 6a,b). Fruit storage trials demonstrated overexpression of *SlIMP3* in the ‘AC’ tomato delayed fruit softening and decreased the water loss. After 30 d of storage at 25°C the *SlIMP3*‐overexpressed fruits had better visual ratings and less water loss compared to the WT fruits (Figure 6c,d) demonstrating that overexpression of *SlIMP3* delayed fruit softening. Fruit firmness at four different developmental stages, mature green, breaker, orange, and red demonstrated firmness gradually decreased with ripening in both WT and *SlIMP3*‐overexpressed fruits. Firmness of the *SlIMP3*‐overexpressed fruit was significantly higher than WT fruit in all four stages, consistent with ‘Micro‐Tom’ fruit results (Figure 6e). The TEM results again demonstrated increased cell wall thickness in the *SlIMP3*‐overexpressed fruits versus WT green fruits (Figure 6f,g). Moreover, this phenotype was also observed when the expression of *SlIMP3* was increased in the ‘AC’ tomato leaves and stems (Figure S11). Consistent with the microtome phenotype, up‐regulation of *SlIMP3* also did not affect the fruit development and maturation (Table S1).

### Increased AsA contents by overexpression of *SlDHAR* gene does not increase cell wall thickness and delay fruit softening in AC tomato

**Figure 7:**
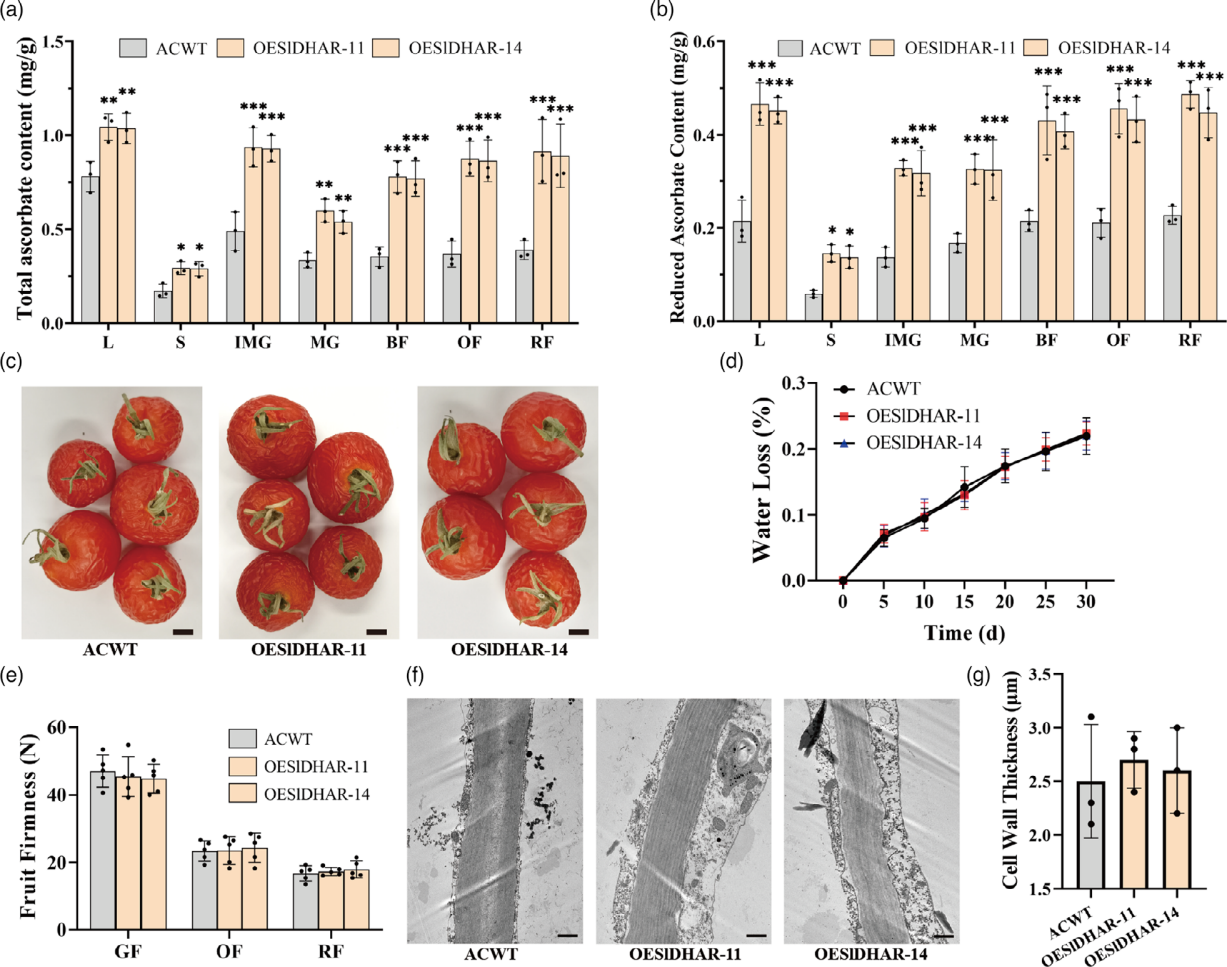
Overexpression of *SlDHAR* gene does not increase cell wall thickness and delay fruit softening. (a, b) Total ascorbate and reduced ascorbate contents in different tomato tissues (L leaf, S stem, IMG immature green fruit, MG mature green fruit, BF break fruit, OF orange fruit, RF red fruit) of ACWT and OESlDHAR lines. (c) Red fruits of ACWT and OESlDHAR lines were stored at 25°C for 30 days. Scale bars, 5 mm. (d) Water loss rate of ACWT and OESlDHAR lines. Statistics of water loss rate every five days. (e) Firmness of fruits at different developmental stages: GF green fruit, OF orange fruit, RF red fruit. (f, g) The difference in cell wall thickness between ACWT and OESlDHAR lines. The cell wall of fruit pericarp cells was observed by transmission electron microscope, and cell wall thickness was measured with ImageJ software. The data contain three replicates and SD was given by error bar. Scale bars, 1 μm. All data contain three replicates and SD was given by error bar. Statistical significance between ACWT and OESlDHAR lines was indicated by asterisk (*P < 0.05, **P < 0.01, ***P < 0.001).

The dehydroascorbate reductase (DHAR) gene plays a key role in the recycling of AsA. To determine if increased AsA biosynthesis increases cell wall thickness and delays fruit softening, the *SlDHAR*‐overexpressed plants were generated in ‘AC’ tomato. Two overexpression lines, OESlDHAR‐11 and OESlDHAR‐14 which exhibited high expression levels of the *SlDHAR* gene, were selected for further analysis (Figure S12). The total and reduced AsA contents were measured in the leaves and stems, and in the different developmental fruit stages. The AsA contents were markedly increased in *SlDHAR‐*overexpressed plants versus the WT plants (Figure 7a,b). However, after 26 days of storage at 25°C, both the WT and *SlDHAR*‐overexpressed fruits were shrivelled (Figure 7c) and had approximately equal water losses (Figure 7d). The fruit firmness in both WT and *SlDHAR*‐overexpressed fruits decreased gradually with ripening and with no significant differences at the different developmental stages (Figure 7e). The TEM observations of fruit cell wall thickness detected no difference between the WT and *SlDHAR*‐overexpressed fruits (Figure 7f,g). These collective results demonstrated overexpression of *SlDHAR* in tomato fruits increased AsA accumulation, but did not affect cell wall biogenesis and fruit softening.

## Discussion

### SlIMP3 functions as a bifunctional enzyme involving in the biosynthesis of AsA and myoinositol

In Arabidopsis, the VTC4 gene (At3g02870) encodes a bifunctional enzyme that catalyses conversion of L‐Gal 1‐P to L‐galactose in AsA biosynthesis and catalyses conversion of the D‐Ins 3‐P to myoinositol(Torabinejad *et al*., 2009). In tomato, three SlIMP isoforms are lithium‐sensitive enzymes that catalyse the myoinositol biosynthesis from myoinositol monophosphate. In this study, the *SlIMP3* gene that had highest expression level among the *SlIMP* genes was expressed and the SlIMP3 protein purified. The SlIMP3 showed high affinity with the L‐Gal 1‐P and D‐Ins 3‐P, and was sensitive to lithium, consistent with enzymatic properties of the Arabidopsis VTC4 (Figure 2). Overexpression of the *SlIMP3* gene increased the AsA content, while downregulation of the *SlIMP3* gene decreased the AsA content in tomato (Figure 2). Overexpression of the *SlIMP3* gene also increased the myoinositol content in tomato fruit (Figure 4). Our data indicated the SlIMP3, which functions like the VTC4, is involved in the biosynthesis of AsA and myoinositol in tomato. But downregulation of the *SlIMP3* did not decrease the myoinositol in tomato fruit (Figure 4), which may indicate a redundancy in myoinositol biosynthesis, and suggests the SlIMP1 and SlIMP2 may supplement the enzymatic activity of SlIMP3 in tomato. Interestingly, we found that the expression level of *SlIMP2* was significantly up‐regulated in *SlIMP3*‐downregulated fruits, which may compensate myoinositol biosynthesis. However, the relationship between *SlIMP2* and *SlIMP3* remains unknown. Perhaps, in future studies, a tertiary mutant of SlIMPs can be generated using CRISPR/Cas9 methods and used to study their precise function in the biosynthesis of AsA and myoinositol in tomato.

In Arabidopsis, overexpression of the myoinositol oxygenase (MIOS) increased AsA content, suggesting that myoinositol can act as a precursor for ascorbate biosynthesis(Lorence *et al*., 2004). However, it has also been reported that the MIOS controls the myoinositol level, but does not contribute to AsA biosynthesis (Endres and Tenhaken, 2009). In this study, treating fruits with 100 mg/l of myoinositol not only increased the cell wall biogenesis, but also increased the fruit’s AsA content (Figure 4). The myoinositol was converted into D‐Glucuronic acid by MIOS and the D‐Glucuronic acid was catalysed into L‐gulonate, which can be converted into the AsA by a multistep process in animals (Bashline *et al*., 2014). Our results support that the hypothesis that the myoinositol oxidation pathway contributes to AsA biosynthesis in tomato fruit. Perhaps rigorous radiotracer experiments could elucidate the pathway of myoinositol conversion into AsA in tomato fruits.

### Increased myoinositol biosynthesis, not AsA, improves cell wall biogenesis and delays fruit softening

Myoinositol has been reported to play an important role in cell wall formation (Loewus, 2006). Myoinositol is catalysed by inositol oxidase to D‐glucuronic acid, a precursor of pectin and hemicellulose in cell wall biosynthesis (Loewus *et al*., 1962; Rigano *et al*., 2018; Yang *et al*., 2021). Radioactive myoinositol injected into ripening strawberry fruits was converted to the D‐galacturonosyl residue of pectin and D‐xylosyl residues of hemicellulose (Loewus *et al*., 1962). In this study, overexpression of *SlIMP3* markedly increased the myoinositol, uronic acid and neutral sugar content, and fruit cell wall thickness (Figures 3 and 5). Simultaneously, treating fruits with 100 mg/L of myoinositol produced a similar phenotype as that of *SlIMP3‐*overexpressed fruits (Figure 4). The data presented here proved that myoinositol participates in tomato fruit cell wall biogenesis.

Silencing of the *PL* gene reinforced the tricellular junction in the fruit cell wall and delayed the tomato fruit softening (Uluisik *et al*., 2016). Mutations in the GA2‐oxidase gene improves cutin and wax biosynthesis and increases tomato fruit firmness and shelf‐life (Li *et al*., 2020). In this study, overexpression of *SlIMP3* gene increased cell‐wall thickness and fruit firmness, delayed fruit softening, and prolonged tomato fruit shelf‐life (Figure 3). These results support that improving cell wall biogenesis is an effective strategy for delaying fruit softening and extending fruit shelf‐life. The *SlIMP3* has potential as an important candidate gene for molecular breeding with the goal of improving shelf‐life of tomato fruit. Interestingly, treating tomato fruits with myoinositol also improved cell wall biogenesis, delayed softening, and extended shelf‐life in fruits. Litchi fruit lost less water after myoinositol treatment (Bhushan *et al*., 2019). Collectively, all these results demonstrate that myoinositol has strong potential for improving tomato postharvest life.

Does the increased AsA biosynthesis contribute to the cell wall biogenesis and delayed fruit softening? The *SlDHAR* gene, that is responsible for recycling of AsA, was overexpressed in AC tomato. The *SlDHAR‐*overexpressed plants exhibited higher AsA contents compared with the WT plants. However, cell wall thickness, fruit firmness, wall loss, and softening time in the *SlDHAR‐*overexpressed plants were similar to those in WT plants (Figure 7). The data presented here corroborates that the increased AsA content does not delay softening and or prolonged shelf‐life in tomato fruit.

### Increased cell wall thickness confers fruit resistance to *B. cinerea*

*B. cinerea* is a necrotrophic fungal pathogen leading to grey mould rot. It is among the most destructive postharvest pathogens of fruit (Blanco‐Ulate *et al*., 2016a, 2016b; Li *et al*., 2018). Causing huge economic losses, genetic modification has been attempted to control this postharvest pathogen. The cell wall is an important barrier to pathogen infections (Cantu *et al*., 2008). Simultaneous downregulation of *PG* and *Exp1* genes in tomato fruit reduced cell wall breakdown and susceptibility to *B. cinerea* (Cantu *et al*., 2008). Suppression of *SlPL* in tomato resulted in increased fruit firmness and reduced susceptibility to *B. cinerea* (Yang *et al*., 2017). Overexpression of the carbohydrate‐binding module of expansin 1 (CBM‐SlEXP1) in tomato increased fruit firmness and decreased susceptibility to *B. cinerea* (Perini *et al*., 2017). In this study, overexpression of *SlIMP3* increased cell wall thickness and improved fruit resistance to *B. cinerea* (Figure 3, 5). The increased cell wall thickness serves to retain inhibition of *B. cinerea* infection. Our data corroborated that the cell wall modification is an effective strategy for improving fruit tolerance of postharvest pathogens. In addition, the increased cell wall thickness was not only found in *SlIMP3‐*overexpressed fruit, but also in *SlIMP3‐*overexpressed leaf and stem (Figure S10, S12). Therefore, we speculate that *SlIMP3* may confer resistance to broad‐spectrum pathogens in tomato.

In conclusion, SlIMP3 is a bifunctional enzyme with the ability to regulate AsA and myoinositol biosynthesis. Overexpressing *SlIMP3* in tomato not only increased AsA accumulation, but also delayed the fruit softening and enhanced tolerance to *B. cinerea*, suggesting the potential value of SlIMP3 in plant improvement programmes with the goal of improving postharvest fruit life.

## Materials and methods

### Plant materials and growth conditions

The tomato (Solanum lycopersicum) cultivars ‘Micro‐Tom’ and ‘Ailsa Craig’ were cultivated in the standard greenhouse conditions; a 14‐h‐day/10‐h‐night cycle, 25°C/18°C day/night temperature, 60% relative humidity, and 250 mol/m^2^/s intense light.

### Plasmid construction and generation of transgenic plants

*SlIMP3* promoter and the full‐length *SlIMP3* coding sequence were amplified using tomato genomic DNA and cDNA, respectively. PCR primers used for amplification were detailed in Table S2. For construction of GUS staining vector, the *SlIMP3* promoter sequence was ligated into the pLP100 containing *GUS* reporter gene. For construction of the overexpression vector, the coding sequences of *SlIMP3* and *SlDHAR* were ligated into binary vector pLP100 containing cauliflower mosaic virus (CaMV) 35S promoter. For antisense vector, the *SlIMP3* coding sequence was inserted into the pLP100‐35S vector in antisense orientation. The constructions were transferred into Agrobacterium strain GV3101 and *Agrobacterium tumefaciens*‐mediated transformation was performed to obtain transgenic lines according to the method described by Wu *et al*. (2020). The transgenic plants were verified by qRT‐PCR with primers listed in Table S2. All experiments in this paper were performed using homozygous lines from T3 generation of the transgenic plants.

### GUS staining and analysis

Fresh tomato tissues from the transgenic plants containing the *SlIMP3* promoter‐GUS construct were harvested and then placed in GUS staining solution (0.1 M sodium phosphate buffer, pH 7.2, 10 mm of EDTA). The tissues were vacuumed for 15 min twice, and incubated in the solution at 37°C for 12 h. After that, the tissues were dipped in graded ethanol series and observed under a light microscope.

### Expression of recombinant protein and phosphatase activity assays

The *SlIMP3* coding sequence was inserted the pGEX‐4T‐1 vector to construct a prokaryotic expression plasmid. The recombinant expression vector (pGEX‐4T‐1‐GST‐SlIMP3) was transformed into E. coli strain Rosetta (DE3). The protein was induced by 0.5 mm of IPTG (Isopropyl‐beta‐D‐thiogalactopyranoside) for 6 h at 28°C, purified by affinity chromatography, and identified by SDS‐PAGE.

SlIMP3 activity was determined according to the method described by Torabinejad *et al*. (2009). A 50 μL of mixture with 50 mm of Tris–Cl, pH 7.0, 3.5 mm of MgCl_2_, 0.5 mm of substrate, and 2 mg of purified SlIMP3 proteins were used as the standard reaction system. The mixture was incubated at 25°C for 30 s to 10 min, and then 800 μL of malachite green reagent was added to stop the reaction. Protein concentrations were determined using a BCA kit (Beijing Dingguo Changsheng BioTechnologies co. Ltd.). GraphPad Prism 8 software was used to analyse kinetic experimental data.

### qRT‐PCR

A total RNA tissue sample was isolated using the RNeasy plant mini kit (Qiagen) and reverse transcribed into cDNA by the HiScript II Q Select RT SuperMix (Vazyme Biotech). qRT‐PCR was conducted using All‐in‐OneTM qPCR mix (GeneCopoeia) according to Wu *et al*. (2020). The relative expression level for each gene was calculated using the ΔΔCt values with *Actin* and *Ubiquitin* as internal controls. The primer sequences used were listed in Table S2. qRT‐PCR was carried out with three biological replicates and each replicate sample was taken from different tomato plants.

### AsA and ethylene measurements

Total and reduced AsA contents were measured according to the method described by Torres‐Contreras *et al*. (2017). For ethylene analysis, ethylene production from different developmental stages of fruits were measured by Agilent 7820A gas chromatograph (Agilent, Santa Clara, CA) as previously described(Liu *et al*., 2014). The measurement was carried out with three biological replicates and each replicate sample was taken from different tomato plants.

### Fruit firmness analysis and shelf‐life analysis

Fruit firmness was measured using a GY‐4 digital fruit sclerometer (Aiwoshi, China). For each line, 20 fruits were collected for measurement. To evaluate the fruit postharvest life, red fruits (breaker + 7) were kept at 25°C for 20–30 days. Individual fruit resh weight was measured to calculate percentage water loss every 2 days. Visual softening and fruit deterioration were evaluated as previously described (Nambeesan *et al*., 2010). Four groups (total 24 fruits) were harvested from each line for shelf‐life analysis.

### Cell wall anatomy analysis

For semi‐thin sections, the leafs, stems, and fruit pericarp were fixed in 4% glutaraldehyde, dehydrated in a series of ethanol baths, and embedded in epoxy resin. The tissues were sectioned at 0.5 μm with a microtome, stained with 1% methylene blue, and observed with light microscopy. The cell wall thickness was measured using ImageJ software. For transmission electron microscopy, the cell walls of the pericarp tissues were examined with a FEI Tecnai T12 twin transmission electron microscope based on the method of Nguyen *et al*. (2014).

### Myoinositol analysis

Myoinositol content was measured using the method described by Endres and Tenhaken (2009). Tissue was ground in liquid nitrogen and extracted with a mixture of MeOH, chloroform, and water (v/v/v, 101:4:4). The sample was centrifuged and the supernatant extracted with a mixture of chloroform and water (v/v, 9:23). The sample was centrifuged, and the supernatant dried and resuspended in water. The sample was centrifuged again and the supernatant used for HPLC measurements with an ICS3000 system (Dionex). A CarboPac MA1 analytical column (Dionex) was used with 120 mm of NaOH as the isocratic eluent at a flow rate of 0.4 mL min^−1^. The measurement was performed with three biological replicates; each replicate sample was taken from different plant.

### Cell wall content analysis

The fruit pericarp samples were homogenized using 95% ethanol and boiled in a water bath for 45 min. After centrifuging, the residue was washed with boiling ethanol, chloroform–methanol (v/v, 1:1), and acetone in sequence. The residue was dried at 25°C and named as alcohol insoluble residue (AIR). Measurement of the uronic acid was determined following the method of (Ahmed and Labavitch, 1978). Five mg of AIR was mixed with 2 mL of concentrated H_2_SO_4_, and water added to the solution until the AIR was completely dissolved. Absorbance was measured at 520 nm and calculated using a galacturonic acid standard curve.

Neutral sugars were measured using the methods described earlier (Nguyen *et al*., 2014). Gas chromatography‐mass spectrometry (GC‐MS) analysis was performed with an Agilent GC‐MS‐HP6890. The various neutral sugars were confirmed based on peak areas and calculated based on standard curves with D‐chiro‐inositol as the internal standard. The measurement was performed with three biological replicates and each replicate sample was taken from different plants.

### *B*.*cinerea* infection

The spores of *B. cinerea* were collected with 0.1% (v/v) tween‐20 solution after cultivation on a PDA medium at 25°C for 15 days. The red fruits (breaker+7 days) of the WT and transgenic plants were harvested, the fruit epidermis was stabbed using a needle. After 10 μL of spore suspension (5 × 10^5^ spores/mL) was dropped on the wound area of fruits were kept at 25°C and high humidity. Lesion diameter was measured 24, 48, and 72 h after inoculation. At 72 h after inoculation, a 1 cm^3^ of fruit sample was collected from around the wound area genomic DNA extraction. The biomass of *B. cinerea* was determined by qRT‐PCR.

### Statistical analysis

All experiments in our study were repeated at minimum of three times. All data was analysed using SPSS software version 21.0. The Student’s t‐test was used to analyse the difference between control and treatment groups. We designated a P value less than 0.05 as a significant difference.

## Conflicts of interest

The authors declare no competing interests.

## Author contributions

W.D., B.Z.Z., Z.G.L., and L.F. planned and designed the research. X.Z.Z., Y.J.Y., B.W.H., X.W.H., Y.W.T., X.X., M.B.W., Z.H.G., Y.Q.L., M.G., X.L.G., G.L.W., Q.D.Z., L.Z., and H.C. performed experiments and analysed the data. X.Z.Z., Y.J.Y., W.D., L.Z., and L.F. wrote the manuscript.

## Supporting information


**Figure S1** Synthetic pathways of myoinositol and AsA in plants.
**Figure S2** Differences in relative expression level of SlIMP1, SlIMP2, and SlIMP3 in various tomato tissues and stages.
**Figure S3** SDS‐PAGE analysis of SlGPP proteins.
**Figure S4** Relative expression level of genes related to AsA metabolism in overexpression (OESlIMP3‐27, OESlIMP3‐28) and antisense (ASSlIMP3‐2, ASSlIMP3‐3) lines.
**Figure S5** Content of ethylene of WT and OESlIMP3/ASSlIMP3 lines fruits at different developmental stages.
**Figure S6** Changes in the expression level of SlIMP3 do not affect the weight and yield of Micro‐tom tomato fruits.
**Figure S7** Relative expression level of genes related to cell wall metabolism in overexpression (OESlIMP3‐27, OESlIMP3‐28) and antisense (ASSlIMP3‐2, ASSlIMP3‐3) lines.
**Figure S8** Relative expression level of SlIMP1 and SlIMP2 in overexpression (OESlIMP3‐27, OESlIMP3‐28) and antisense (ASSlIMP3‐2, ASSlIMP3‐3) lines.
**Figure S9** Overexpression of SlIMP3 increased cell wall thickness of leaf and stem in ‘Micro‐tom’ tomato.
**Figure S10** Relative expression level of SlIMP3 in overexpression (OESlIMP3‐1, OESlIMP3‐9) lines.
**Figure S11** Overexpression of SlIMP3 increased cell wall thickness of leaf and stem in ‘Ailsa Craig’ tomato.
**Figure S12** Relative expression level of SlDHAR in overexpression (OESlDHAR‐11, OESlDHAR‐14) lines.
**Table S1** Breaking time and maturation time of all tomato lines.
**Table S2** Primer sequences of genes mentioned in this article.
