# Supplementary material for: Control of fruit softening and Ascorbic acid accumulation by manipulation of SlIMP3 in tomato
Source: Plant Biotechnol J. 2022 Mar 15;20(6):1213–25. doi: 10.1111/pbi.13804 (PMC9129080; doi:10.1111/pbi.13804)
Supplement: Supplementary file 1 — Figure S1 Synthetic pathways of myoinositol and AsA in plants. Figure S2 Differences in relative expression level of SlIMP1, SlIMP2, and SlIMP3 in various tomato tissues and stages. Figure S3 SDS‐PAGE analysis of SlGPP proteins. Figure S4 Relative expression level of genes related to AsA metabolism in overexpression (OESlIMP3‐27, OESlIMP3‐28) and antisense (ASSlIMP3‐2, ASSlIMP3‐3) lines. Figure S5 Content of ethylene of WT and OESlIMP3/ASSlIMP3 lines fruits at different developmental stages. Figure S6 Changes in the expression level of SlIMP3 do not affect the weight and yield of Micro‐tom tomato fruits. Figure S7 Relative expression level of genes related to cell wall metabolism in overexpression (OESlIMP3‐27, OESlIMP3‐28) and antisense (ASSlIMP3‐2, ASSlIMP3‐3) lines. Figure S8 Relative expression level of SlIMP1 and SlIMP2 in overexpression (OESlIMP3‐27, OESlIMP3‐28) and antisense (ASSlIMP3‐2, ASSlIMP3‐3) lines. Figure S9 Overexpression of SlIMP3 increased cell wall thickness of leaf and stem in ‘Micro‐tom’ tomato. Figure S10 Relative expression level of SlIMP3 in overexpression (OESlIMP3‐1, OESlIMP3‐9) lines. Figure S11 Overexpression of SlIMP3 increased cell wall thickness of leaf and stem in ‘Ailsa Craig’ tomato. Figure S12 Relative expression level of SlDHAR in overexpression (OESlDHAR‐11, OESlDHAR‐14) lines. Table S1 Breaking time and maturation time of all tomato lines. Table S2 Primer sequences of genes mentioned in this article. [file PBI-20-1213-s001.docx]

**Supplementary Materials**


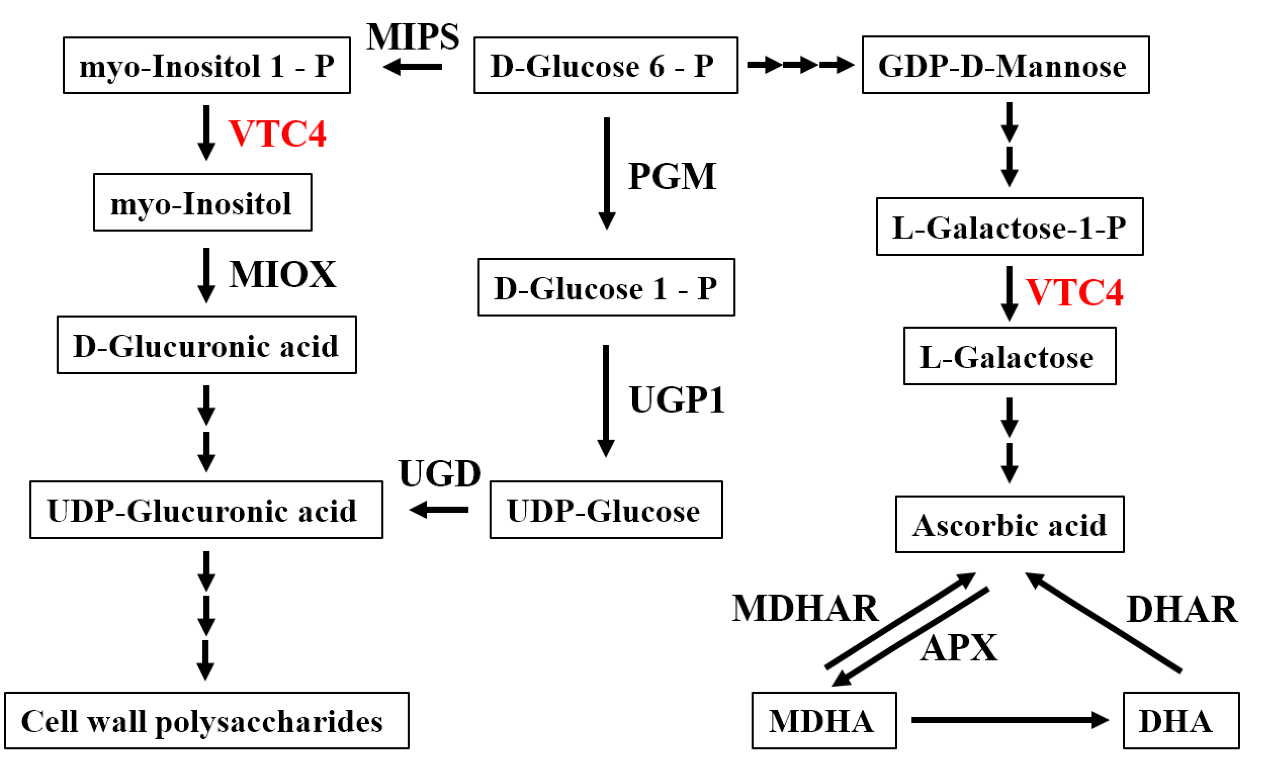


**Figure S1**. Synthetic pathways of myoinositol and AsA in plants.

The red font indicates that VTC4 was involved in the biosynthesis of myoinositol and AsA simultaneously. MIPS: myoinositol phosphate synthase; VTC4: L-galactose-1-phosphate phosphatase; MIOX: myoinositol oxygenase; PGM: phosphoglucosemutase; UGP1: UTP-Glc-1-P uridylyltransferase; UGD: UDP-Glc dehydrogenase; APX: Ascorbate peroxidase; MDHA: Monodehydroascorbic acid; MDHAR: Monodehydroascorbate reductase; DHA: Dehydroascorbic acid; DHAR: Dehydroascorbate reductase.


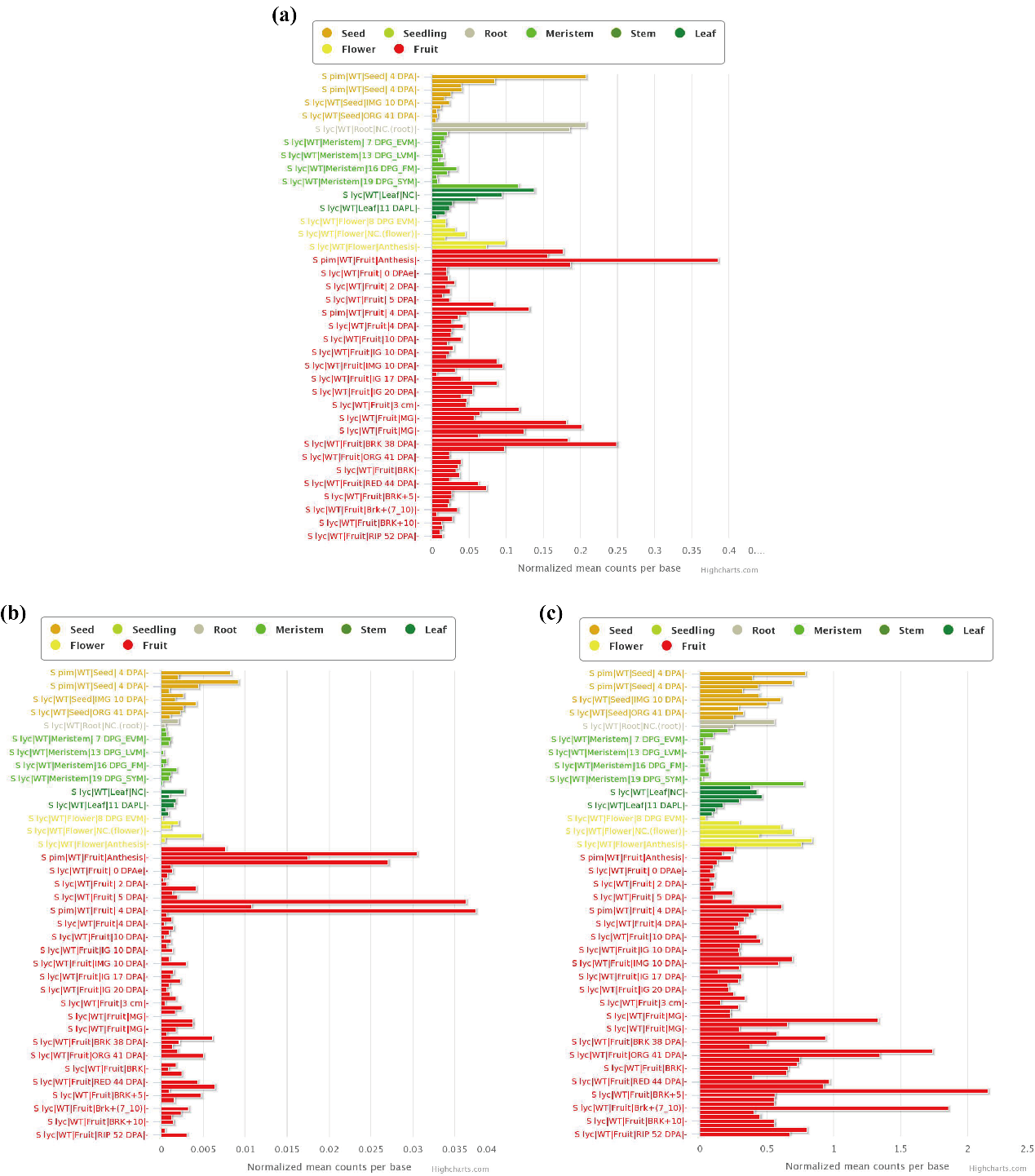


**Figure S2.** Differences in relative expression level of *SlIMP1*, *SlIMP2* and *SlIMP3* in various tomato tissues and stages

**a,** The relative expression level of *SlIMP1* in various tomato tissues and stages.

**b,** The relative expression level of *SlIMP2* in various tomato tissues and stages.

**c,** The relative expression level of *SlIMP3* in various tomato tissues and stages.

Data was downloaded from http://tomexpress.toulouse.inra.fr/


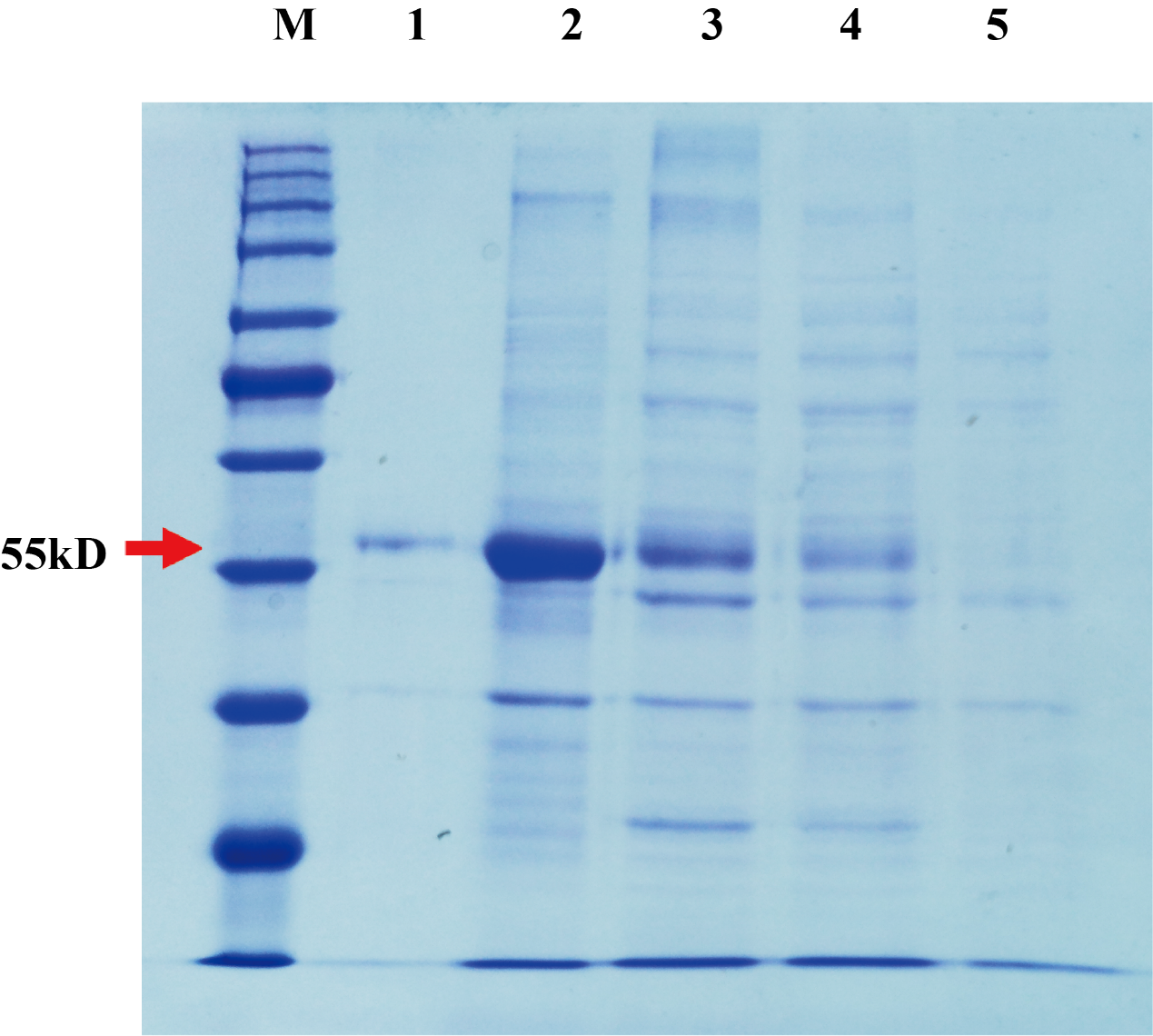


**Figure S3.** SDS-PAGE analysis of SlGPP proteins. SlGPP proteins were produced by prokaryotic expression. M, marker. 1, non-induced. 2, induced with 0.5 mM IPTG for 6 h at 28 ℃. 3, induced with 1 mM IPTG for 6 h at 28 ℃. 4, induced with 0.5 mM IPTG for 3 h at 37 ℃. 5, induced with 1 mM IPTG for 3 h at 37 ℃. The protein band of SlGPP was indicated by a red arrow


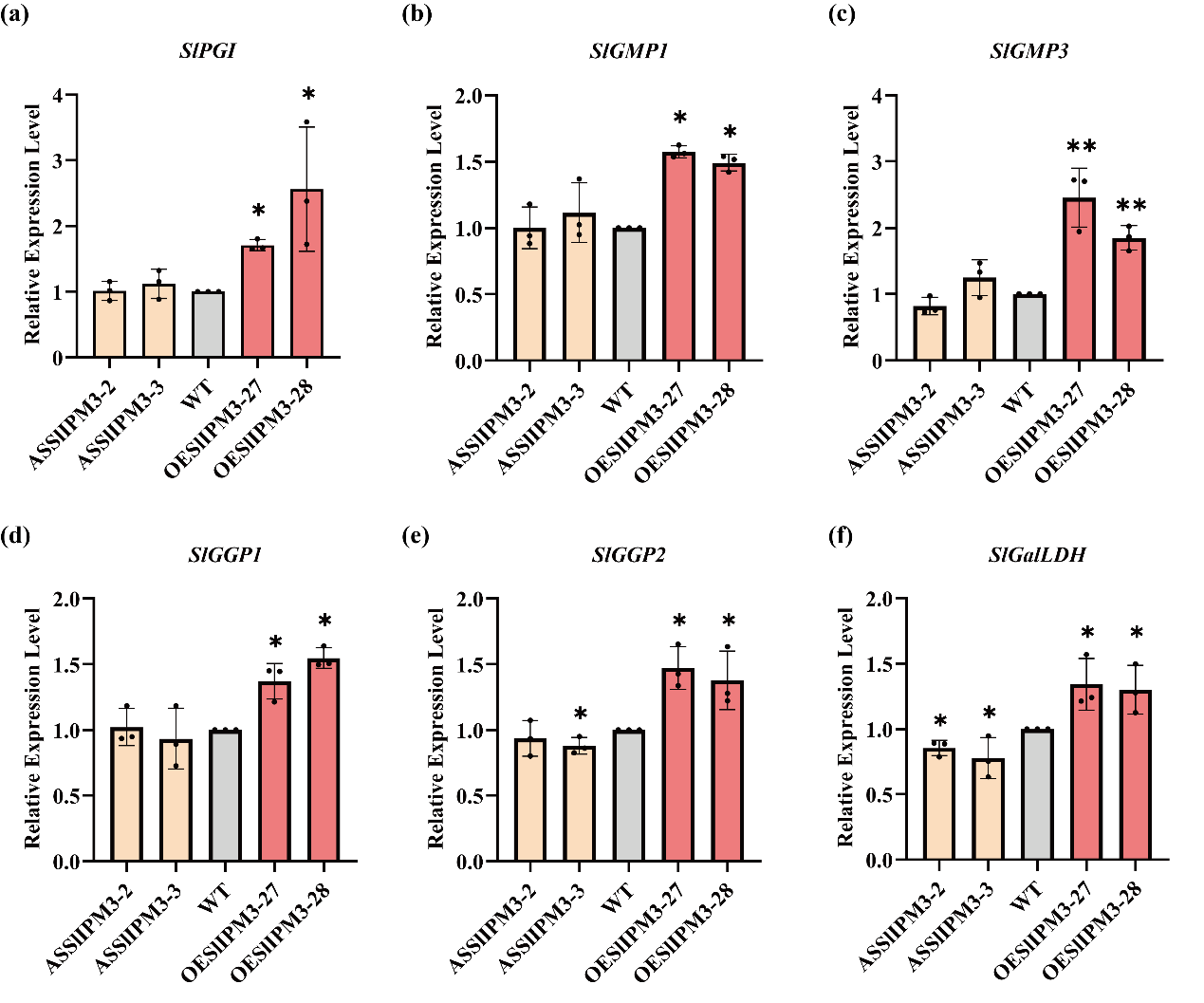


**Figure S4.** Relative expression level of genes related to AsA metabolism in overexpression (OESlIMP3-27, OESlIMP3-28) and antisense (ASSlIMP3-2, ASSlIMP3-3) lines. All data contains three replicates, and error bar represents SD. Statistical significance between WT and OESlIMP3/ASSlIMP3 lines was indicated by asterisk (*P < 0.05, **P < 0.01).





**Figure S5.** Content of ethylene of WT and OESlIMP3/ASSlIMP3 lines fruits at different developmental stages: MG mature green fruit, BR + 2 break + 2 days fruit, BR + 3 break + 3 days fruit, BR + 5 break + 5 days fruit, BR + 12 break + 12 days fruit, BR + 20 break + 20 days fruit. All data contains three replicates and SD was given by error bar.


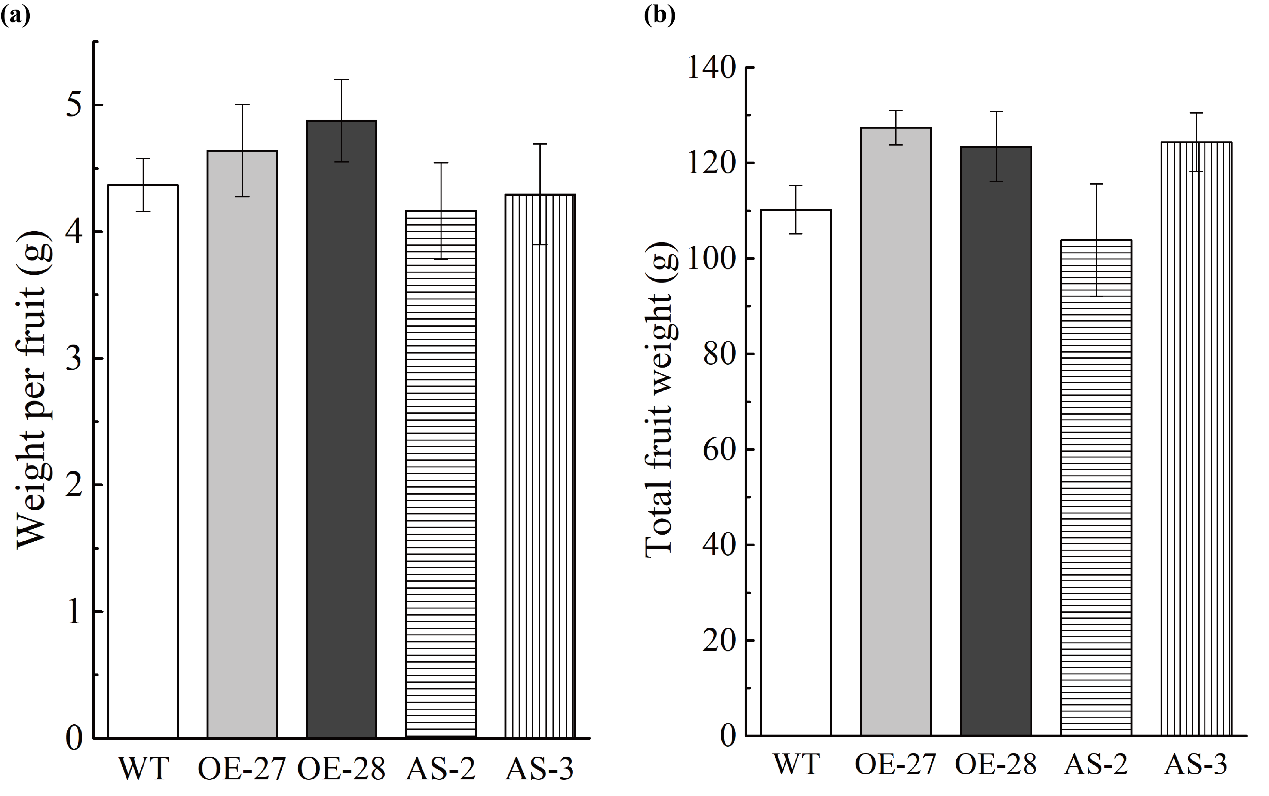


**Figure S6.** Changes in the expression level of *SlIMP3* do not affect the weight and yield of Micro-tom tomato fruits.

**a,** The weight of a single red fruit of WT and OESlIMP3/ASSlIMP3 lines.

**b,** The total red fruits weight of WT and OESlIMP3/ASSlIMP3 lines.

All data contains three replicates, and error bar represents SD.


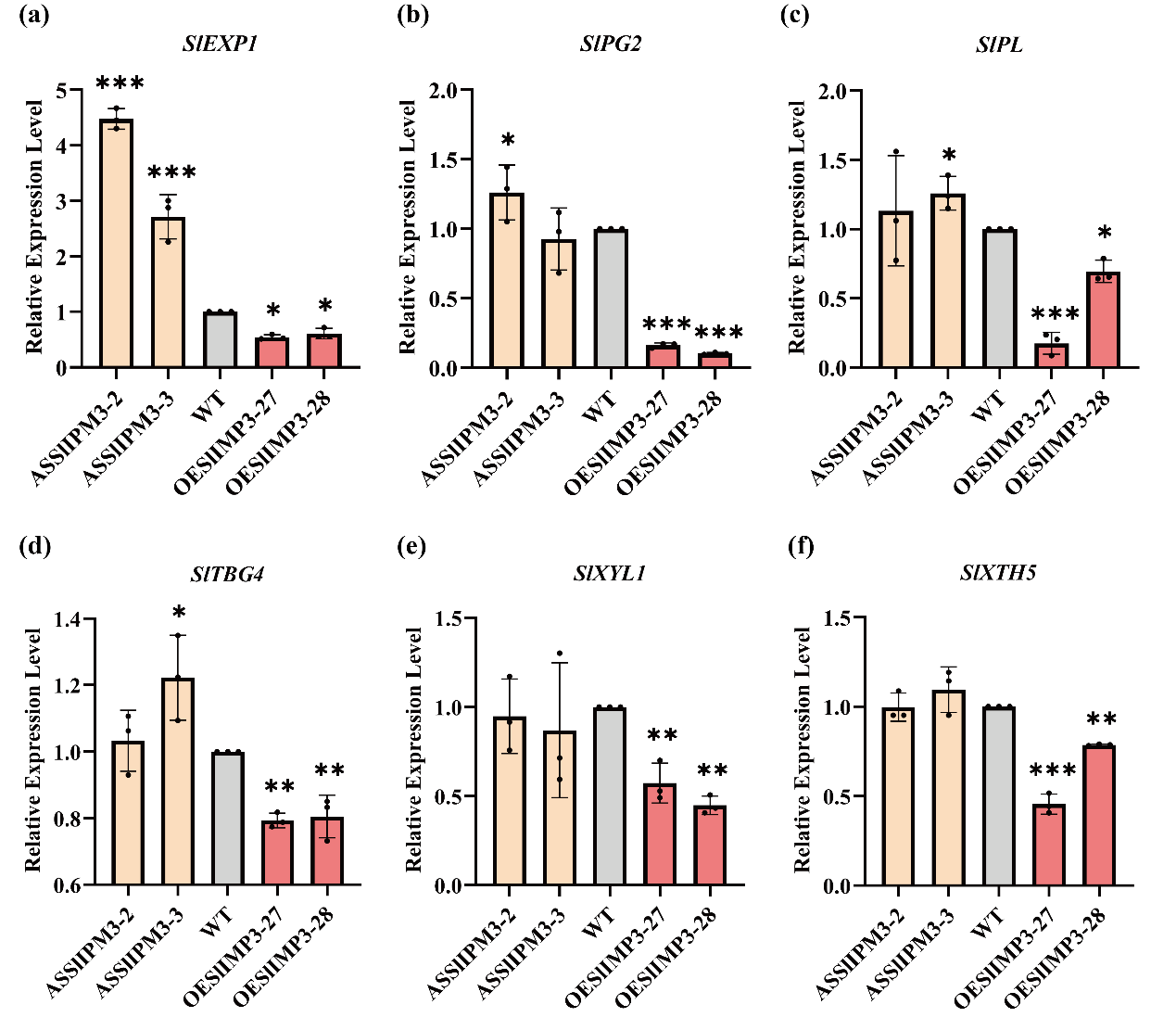


**Figure S7.** Relative expression level of genes related to cell wall metabolism in overexpression (OESlIMP3-27, OESlIMP3-28) and antisense (ASSlIMP3-2, ASSlIMP3-3) lines. All data contains three replicates, and error bar represents SD. Statistical significance between WT and OESlIMP3/ASSlIMP3 lines was indicated by asterisk (*P < 0.05, **P < 0.01, ***P < 0.001).


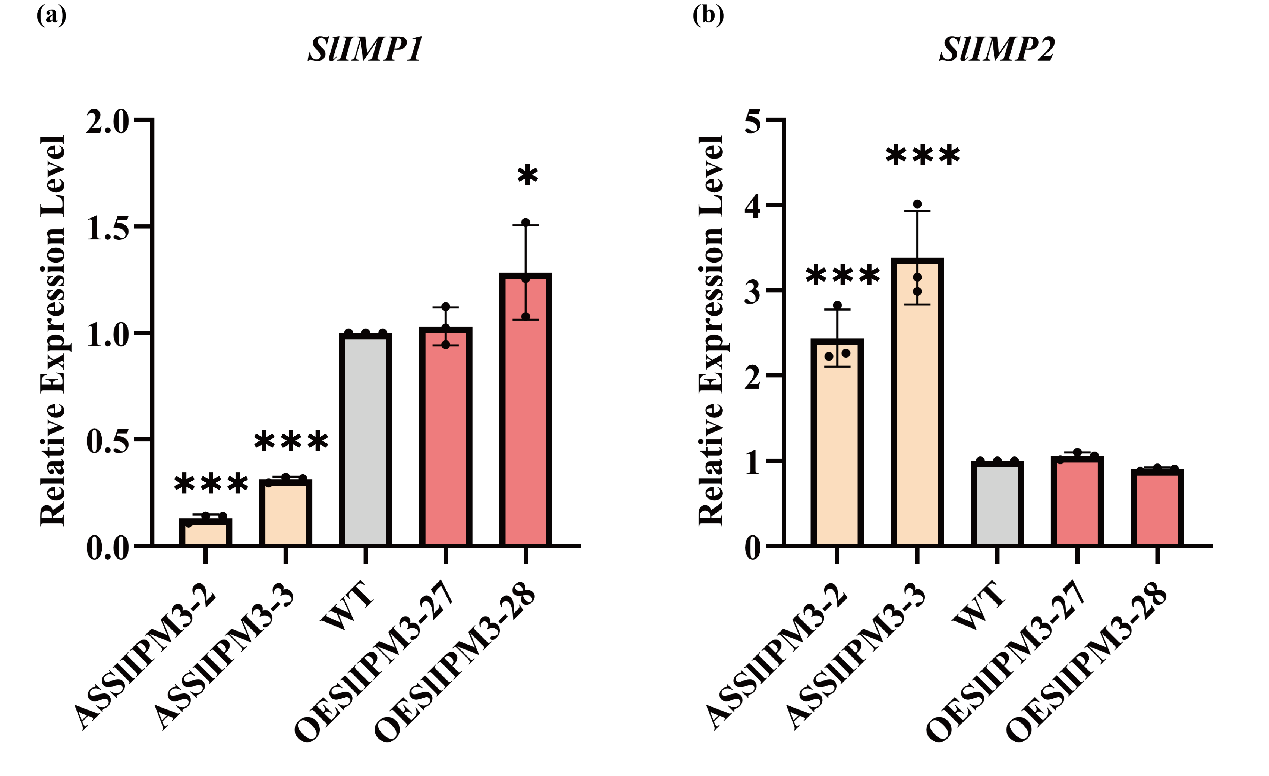


**Figure S8.** Relative expression level of *SlIMP1* and *SlIMP2* in overexpression (OESlIMP3-27, OESlIMP3-28) and antisense (ASSlIMP3-2, ASSlIMP3-3) lines. All data contains three replicates, and error bar represents SD. Statistical significance between WT and OESlIMP3/ASSlIMP3 lines was indicated by asterisk (*P < 0.05, ***P < 0.001).


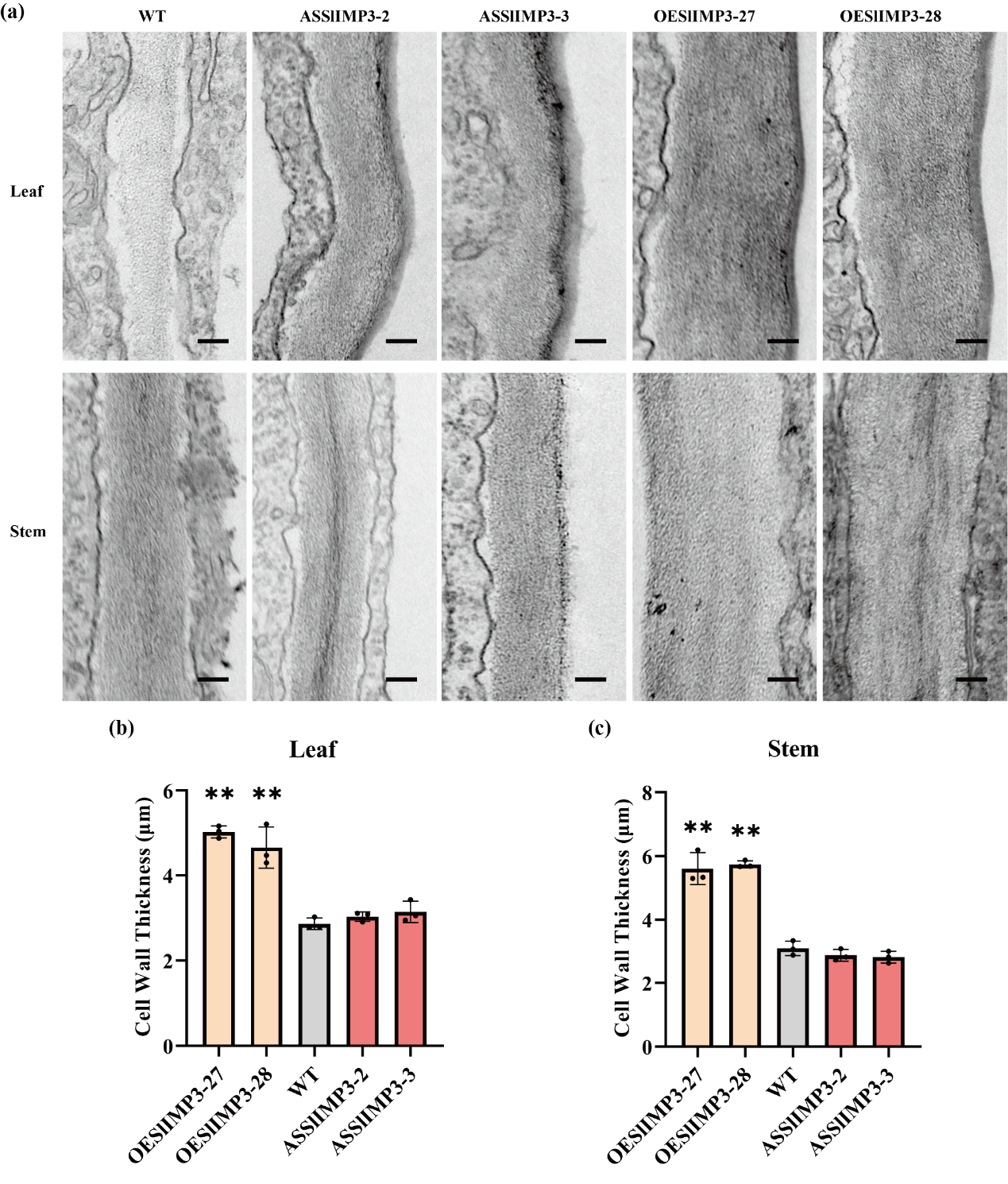


**Figure S9.** Overexpression of SlIMP3 increased cell wall thickness of leaf and stem in ‘Micro-tom’ tomato. **(a, b, c)** The difference in cell wall thickness between WT and OESlIMP3/ASSlIMP3 lines. The cell wall of leaf and stem cells were observed with transmission electron microscope. The cell wall thicknerss was measured with ImageJ software. The data contains three replicates and SD was given by error bar. Scale bars, 1 μm. Statistical significance between WT and OESlIMP3/ASSlIMP3 lines was indicated by an asterisk (**P < 0.01).


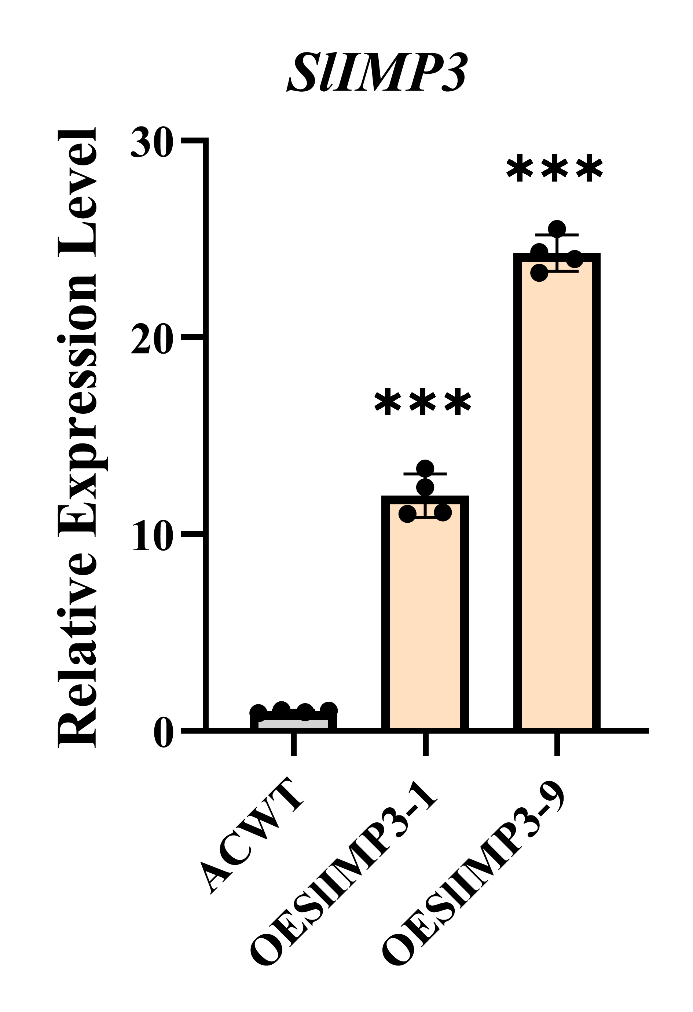


**Figure S10.** Relative expression level of *SlIMP3* in overexpression (OESlIMP3-1, OESlIMP3-9) lines. All data contains three replicates, and error bar represents SD. Statistical significance between ACWT and OESlIMP3 lines was indicated by asterisk (***P < 0.001).


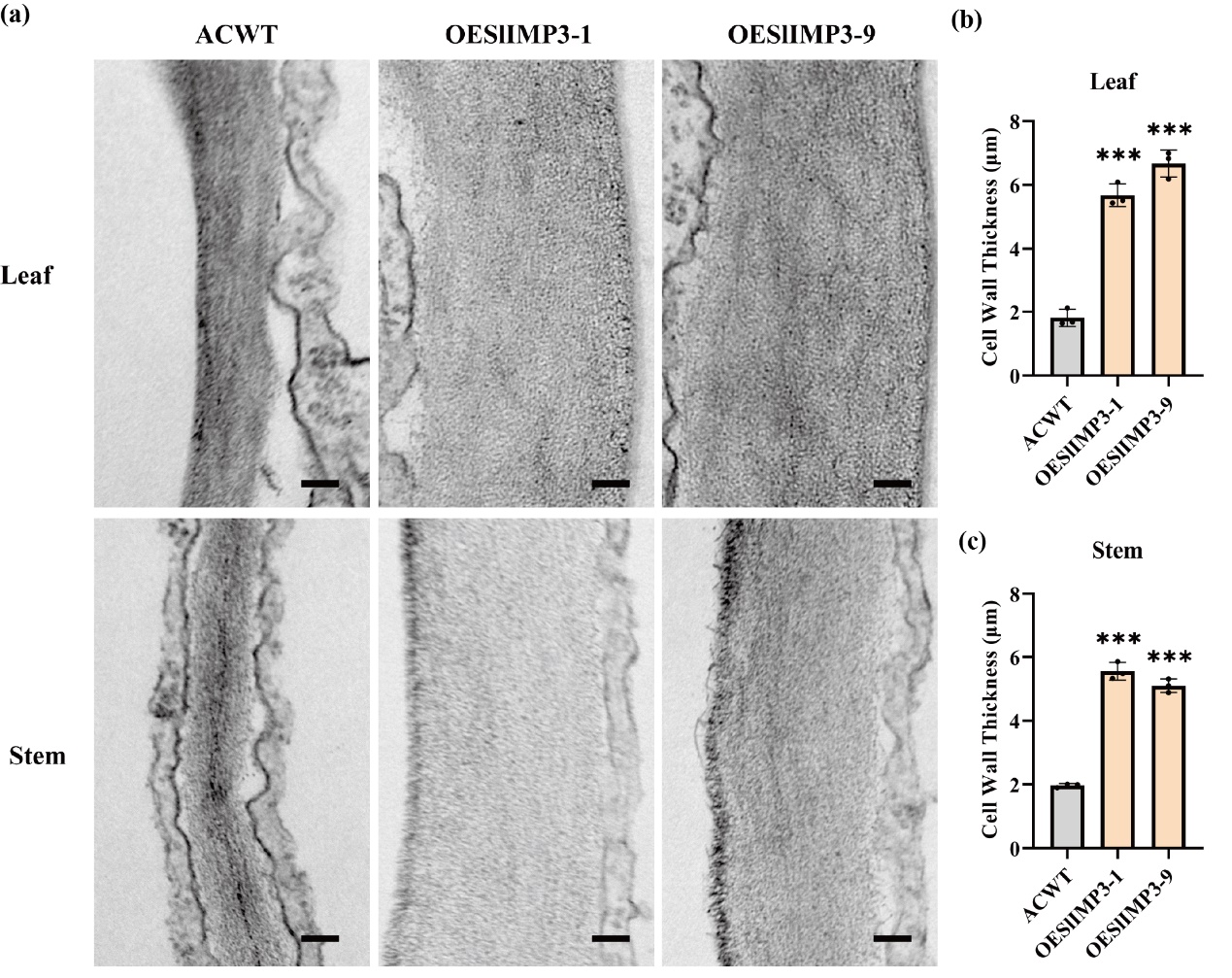


**Figure S11.** Overexpression of SlIMP3 increased cell wall thickness of leaf and stem in ‘Ailsa Craig’ tomato. (a, b, c) The difference in cell wall thickness between ACWT and OESlIMP3 lines. The cell wall of leaf and stem cells were observed with transmission electron microscope. The cell wall thicknerss was measured with ImageJ software. The data contains three replicates and SD was given by error bar. Scale bars, 1 μm. Statistical significance between ACWT and OESlIMP3 lines was indicated by an asterisk (***P < 0.001).


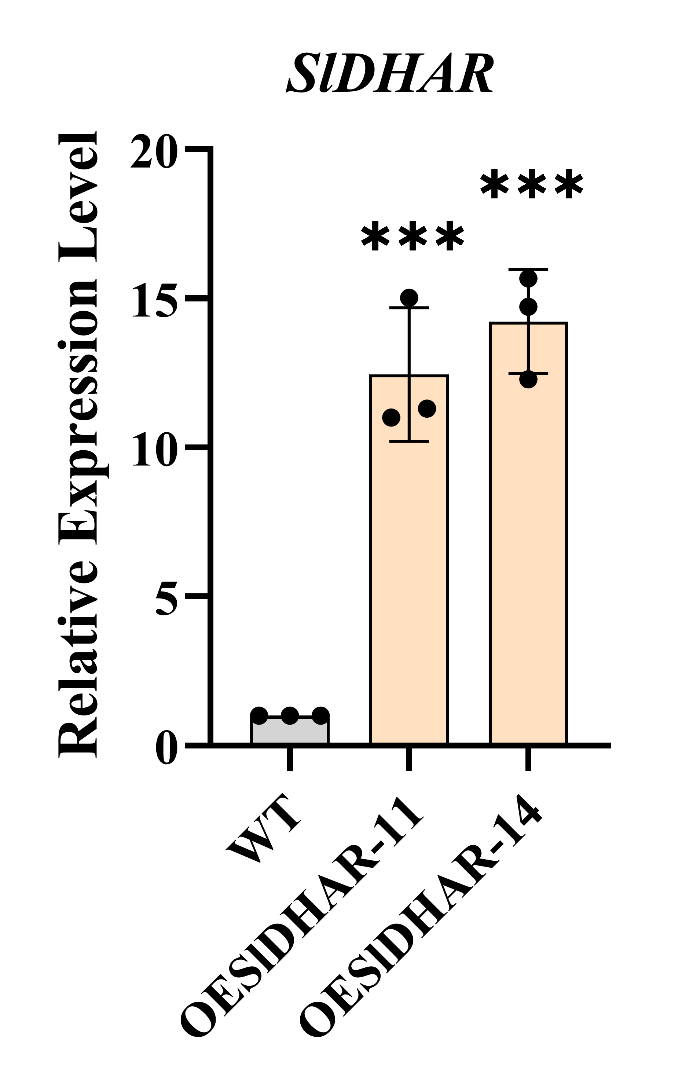


**Figure S12.** Relative expression level of *SlDHAR* in overexpression (OESlDHAR-11, OESlDHAR-14) lines. All data contains three replicates, and error bar represents SD. Statistical significance between ACWT and OESlDHAR lines was indicated by asterisk (***P < 0.001).

**Table S1.** Breaking time and maturation time of all tomato lines.

| Line | Breaking time (DPA) | Maturation time (DPA) |
| --- | --- | --- |
| WT | 35 ± 1 | 42 ± 1 |
| OESlIMP3-27 | 34 ± 1 | 42 ± 1 |
| OESlIMP3-28 | 35 ± 2 | 41 ± 1 |
| ASSlIMP3-2 | 34 ± 1 | 40 ± 2 |
| ASSlIMP3-3 | 35 ± 1 | 41 ± 1 |
| ACWT | 40 ± 1 | 47 ± 1 |
| OESlIMP3-1 | 41 ± 1 | 46 ± 1 |
| OESlIMP3-9 | 40 ± 2 | 47 ± 1 |

**Table S2.** Primer sequences of genes mentioned in this article.

| **Name** | **Sequences (5’→3’)** | **Description** |
| --- | --- | --- |
| SlIMP1-qPCR-F | TTGTGAAGTCCCTCCTTGGC | qRT-PCR |
| SlIMP1-qPCR-R | ACACGAACCACACATTCGGA |  |
| SlIMP2-qPCR-F | AGCTGGAGGACTCGTTCTTG |  |
| SlIMP2-qPCR-R | AGGCGTTGATAAAAGCGTCC |  |
| SlIMP3-qPCR-F | CTACAACAGGGAGAATCAATAGC |  |
| SlIMP3-qPCR-R | TCTTTCACAATCACAGCACCA |  |
| SlDHAR-qPCR-F | CATCTCGAGGTGGCTCTTGG |  |
| SlDHAR-qPCR-R | TTTCTGGAACGACTCTCGCT |  |
| SlPGI-qPCR-F | TGCTCTTCAAAAGCGTGTCC |  |
| SlPGI-qPCR-R | CGGCAATAAGTGCTCTGTCA |  |
| SlGMP1-qPCR-F | TGAAATCAAGGAAAATGCGG |  |
| SlGMP1-qPCR-R | ACCGACACGGATTCACCAAG |  |
| SlGMP3-qPCR-F | AAACCTGAAATCGTGATGTGAGA |  |
| SlGMP3-qPCR-R | TGAAGAAGAGGAGAACTGGAAAC |  |
| SlGGP1-qPCR-F | GAAATCTGGTCTGTTCCTCTGTGA |  |
| SlGGP1-qPCR-R | TTCACACACCAACTCCACATTACA |  |
| SlGGP2-qPCR-F | CTGTTGTCTTGGTTGGAGGTTGT |  |
| SlGGP2-qPCR-R | AGCACAGTCAAAACACCAACAAA |  |
| SlGalLDH-qPCR-F | ATTGAGGTTCCCAAGGACATAG |  |
| SlGalLDH-qPCR-R | ATGTTATTAGATAGGATGCGGTTT |  |
| SlEXP1-qPCR-F | CCCTCCTCGCCCTCACTTT |  |
| SlEXP1-qPCR-R | TCTGATTCCTCCTTGCTTTCG |  |
| SlPG2-qPCR-F | ATACAACAGTTTTCAGCAGTTCAAGT |  |
| SlPG2-qPCR-R | GGTTTTCCACTTTCCCCTACTAA |  |
| SlPL-qPCR-F | GCGATCAGGAGTTAGAACTGG |  |
| SlPL-qPCR-R | AATCCCCTTTTGCTTTGGTT |  |
| SlTBG4-qPCR-F | AAATGGTGAAGGCGTAGGTCG |  |
| SlTBG4-qPCR-R | AGGTTGTCCGCAGTTAGTCTGG |  |
| SlXYL1-qPCR-F | TGATCGGCAATTATGAAGGTATTC |  |
| SlXYL1-qPCR-R | CAGCACATCCTGGCTTGTAAAT |  |
| SlXTH5-qPCR-F | CCACCACCAGAGTGCGAGAT |  |
| SlXTH5-qPCR-R | TTTTCTTAGGATGACGATGTCCG |  |
| SlPR-1a-qPCR-F | GAAGATGTGGGTTGATGAGAAGC |  |
| SlPR-1a-qPCR-R | TTACAAGTTATAAAGTACCACCCG |  |
| SlPR-1b-qPCR-F | AGAGGCCAAGCTATAACTACGC |  |
| SlPR-1b-qPCR-R | AATGAACCACCATCCGTTGTTGC |  |
| BcCutA-F | ATTCCACAATATGGCATGAAATC |  |
| BcCutA-R | ATGTTATCTCATGTTATCTC |  |
| Actin-F | CGGTGACCACTTTCCGATCT |  |
| Actin-R | TCCTCACCGTCAGCCATTTT |  |
| Ubiquitin-F | TCGTAAGGAGTGCCCTAATGCTGA |  |
| Ubiquitin-R | CAATCGCCTCCAGCCTTGTTGTAA |  |
| SlIMP3-pro-F | GGCCCTTTCGTCTTCAAGATGATATTTGTATCTGATAC | ProSlIMP3::GUS vector constract |
| SlIMP3-pro-R | TACCCGGGGATCCTCTAGGGGTGATGGATTGGAAAAG |  |
| SlIMP3-OE-F | AACCCGGGATGGCACAAAATGGTTCAGTTG | plp100-35S-SlIMP3 (OE) vector constract |
| SlIMP3-OE-R | AACCCGGGTTCATTCAAGGCCTTGATA |  |
| SlDHAR-OE-F | AACCCGGGATGGTTGTTGAAGTTTGTG | plp100-35S-SlDHAR (OE) vector constract |
| SlDHAR-OE-R | AACCCGGGAACCTTTGGAGCCCACCC |  |
| SlIMP3-AS-F | AACCCGGGTTCATTCAAGGCCTTGATA | plp100-35S-SlIMP3 (AS) vector constract |
| SlIMP3-AS-R | AACCCGGGATGGCACAAAATGGTTCAGTTG |  |
| SlIMP3-GST-F | GGTTCCGCGTGGATCCATGGCACAAAATGGTTCAGTTG | pGEX-4T-1-GST-SlIMP3 vector constract |
| SlIMP3-GST-R | AGTCACGATGCGGCCGCTTCATTCAAGGCCTTGATA |  |
